# Keratinocyte-induced costimulation of human T cells through CD6 - but not CD2 - activates mTOR and prevents oxidative stress

**DOI:** 10.3389/fimmu.2022.1016112

**Published:** 2022-10-24

**Authors:** Christian Orlik, Karina M. Berschneider, Beate Jahraus, Beate Niesler, Emre Balta, Knut Schäkel, Jutta Schröder-Braunstein, Maria Margarida Souto-Carneiro, Yvonne Samstag

**Affiliations:** ^1^ Institute of Immunology, Section Molecular Immunology, Heidelberg University Hospital, Heidelberg, Germany; ^2^ Institute of Human Genetics, Department of Human Molecular Genetics and nCounter Core Facility, Heidelberg University, Heidelberg, Germany; ^3^ Department of Dermatology, Heidelberg University Hospital, Heidelberg, Germany; ^4^ Department of Rheumatology, Medical Clinic 5, Heidelberg University Hospital, Heidelberg, Germany

**Keywords:** CD6/CD166/CD318, T cell costimulation, T cell metabolism, keratinocytes, non-professional antigen-presenting cells, inflammatory skin diseases, psoriasis, GvHD

## Abstract

In psoriasis and other inflammatory skin diseases, keratinocytes (KCs) secrete chemokines that attract T cells, which, in turn, cause epidermal hyperplasia by secreting proinflammatory cytokines. To date, it remains unclear whether skin-homing T cells, particularly memory T cells, can also be activated by direct cell contact with KCs. In this study, we demonstrated the ability of primary human KCs to activate human memory T cells directly by transmitting costimulatory signals through the CD6/CD166/CD318 axis. Interestingly, despite being negative for CD80/CD86, KCs initiate a metabolic shift within T cells. Blockade of the CD6/CD166/CD318 axis prevents mammalian target of rapamycin activation and T cell proliferation but promotes oxidative stress and aerobic glycolysis. In addition, it diminishes formation of central memory T cells. Importantly, although KC-mediated costimulation by CD2/CD58 also activates T cells, it cannot compensate for the lack of CD6 costimulation. Therefore, KCs likely differentially regulate T cell functions in the skin through two distinct costimulatory receptors: CD6 and CD2. This may at least in part explain the divergent effects observed when treating inflammatory skin diseases with antibodies to CD6 versus CD2. Moreover, our findings may provide a molecular basis for selective interference with either CD6/CD166/CD318, or CD2/CD58, or both to specifically treat different types of inflammatory skin diseases.

## Introduction

Chronic inflammatory skin diseases are primarily driven by T cell-mediated responses. In psoriasis or cutaneous manifestation of graft-versus-host disease (GvHD), autoreactive or alloreactive T cells, respectively, are recruited through chemokines produced by keratinocytes (KCs) and infiltrate the skin (1–3). Upon infiltration of the dermal and epidermal layers of the skin, these effector T cells secrete various proinflammatory cytokines, including IFNγ and IL-17 (4). IL-17 inhibits KC differentiation and induces its proliferation, thus causing key histological features of these diseases, such as epidermal hyperplasia and follicular erythema (2, 5).

A small subset of these T cells develop into precursor memory cells during the initial phase of chronic inflammatory skin diseases. These precursor cells differentiate into several memory subsets, including effector memory (EM), central memory (CM) and tissue-resident memory (TRM) T cells (6). Within peripheral tissues, that is, lesions of chronic skin inflammation, EM and TRM T cells represent the predominant T cell populations (7). Several studies support the role of TRM T cells in the recurrence of chronic skin inflammation under pathological conditions (8, 9).

However, the exact mechanism by which these memory cells are activated remains under investigation. It has been suggested that during the initiation phase of inflammatory skin diseases, effector T cells arise from naïve T cells that are exclusively activated by mature dermal DCs in the skin-draining lymph nodes (10). In this context, primary KCs may also play an important role since they can activate naïve T cells under proinflammatory conditions through direct contact (7), which may occur in wounds or skin barrier defects. The lack of essential ligands for costimulatory receptors on KCs, namely CD80 and CD86, which interact with CD28 on T cells, is partially compensated by costimulatory signals transmitted through interaction of CD58 on KCs with the costimulatory receptor CD2, which drive the differentiation of Th1 cells.

The interaction between ligands for costimulatory receptors on professional antigen-presenting cells (APCs) and the respective costimulatory receptors on T cells, along with the interaction of antigen-bound class II major histocompatibility complexes (MHC) and the T cell receptor (TCR), transmits essential signals for full T cell activation (11). Among the major activation processes regulated by costimulatory signals is the rearrangement of the immune synapse, that is, the contact zone between APCs and T cells (12), through the recruitment of adhesion molecules and the initiation of signaling pathways triggering gene expression and metabolic reprogramming (13–15).

The shift of the metabolic program from catabolic metabolism based on oxidative phosphorylation (OXPHOS) to anabolic metabolism driven by aerobic glycolysis and enhanced glutamine uptake and utilization is a pre-requisite for activated and proliferating T cells (16). Aerobic glycolysis increases the production of lactate, which functions as a rapid energy source (17, 18) but also as a mediator during T cell differentiation (19). Costimulatory signals transmitted by CD28 promote the activation of the PI-3K pathways and the stimulation of the mammalian target of rapamycin (mTOR), which positively regulates glycolysis and the expression of nutrient transporters (20–22). These processes, together with enhanced *de novo* synthesis of other molecular building blocks, such as fatty acids (FAs) and nucleic acids, enable naïve T cells to develop into highly proliferative effector T cells (23).

These processes have been well described for the activation of naïve or effector T cells by professional APCs. However, it remains unclear whether memory T cells, especially TRM and EM, are also activated through costimulation processes by skin-homing DCs or by other non-professional APCs, namely KCs, under proinflammatory conditions.

In this study, we addressed the activation process of human memory T cells by non-professional APCs, especially primary human KCs, in the absence of CD28-mediated costimulation. Our data demonstrate a pivotal role of CD6 during the transmission of CD28-independent costimulatory signals for the regulation of T cell metabolism upon KC-T cell interaction.

## Results

### CD58/CD2-dependent costimulation is necessary for KC-mediated stimulation of naïve but not memory T cells

**Figure 1:**
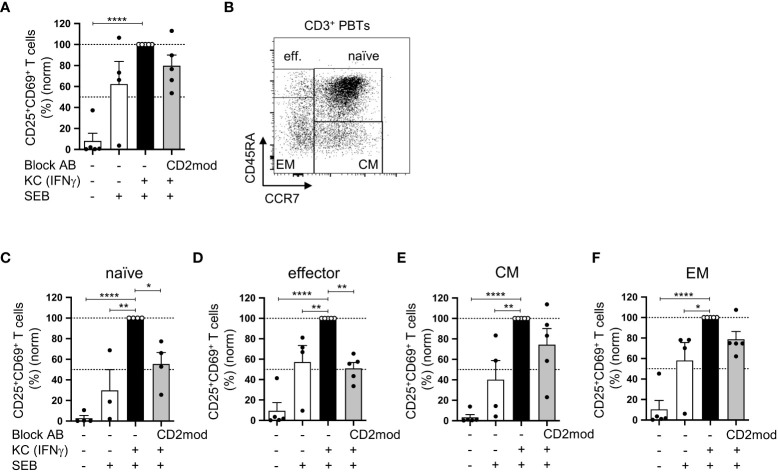
CD58/CD2-dependent costimulation is necessary for KC-mediated activation of naïve and effector T cells but not of memory T cells. CD3^+^ PBTs, either with or without CD2 downmodulation (CD2mod), were cocultured for 24 h with untreated KCs or IFNγ-pretreated KCs loaded with (+) or without (-) SEB. Surface receptor expression was assessed by flow cytometry. **(A)** Statistical evaluation of CD25 and CD69 surface expression after 24 h of coculture (n = 5 individual T cell donors). **(B)** Representative dot plot of CD45RA and CCR7 surface expression on CD3^+^ PBTs. C-F: Statistical evaluation of CD25 and CD69 expression on T cell subpopulations after 24 h coculture (n ≥ 4 individual T cell donors). CD3^+^ PBTs were categorized into **(C)** naïve (naïve, CD45RA^+^CCR7^+^), **(D)** effector (effector, CD45RA^+^CCR7^-^), **(E)** central memory (CM, CD45RA^-^CCR7^+^) and **(F)** effector memory (EM, CD45RA^-^CCR7^-^) PBTs. Data was normalized to PBTs cultured with IFNγ-pretreated KCs loaded with SEB (black bar). Data is represented as mean ± SEM. ****=p<0.0001; **=p<0.01; *=p<0.05.

In a previous study, we established an *in vitro* coculture system that demonstrated the ability of primary human KCs loaded with *staphylococcal* enterotoxin B (SEB) to stimulate human peripheral blood T cells (PBTs) under proinflammatory conditions (7). Therefore, CD58/CD2 interaction is highly important for KC-dependent stimulation of isolated naïve T cells and the subsequent Th1 differentiation process. However, the involvement of CD58/CD2-mediated costimulation in the activation of memory T cells remains unknown. To address this question, PBTs, which include naïve, effector, and memory T cell subsets, were cocultured with IFNγ-pretreated KCs to mimic proinflammatory conditions. The CD58/CD2 interaction was blocked using a CD2-modulating IgM antibody (CD2mod), which selectively downregulated the surface expression of CD2 (**Figure S1**) (7). Similar to our previous study (7), PBTs cocultured with IFNγ-pretreated KCs significantly upregulated the expression of early (CD69) and late (CD25) activation markers compared with PBTs cocultured with unstimulated KCs (**Figure 1A**). Interestingly, unlike naïve cells (6), the upregulated expression of CD25 and CD69 in total PBTs was not significantly reduced by blocking the CD58/CD2 interaction (**Figure 1A**). To determine the influence of CD2-mediated costimulation on the different T cell subsets, particularly on memory T cells, the expression of activation markers was analyzed on PBTs that were discriminated into naïve, effector, and memory T cells according to CD45RA and CCR7 expression (**Figure 1B**). As previously observed, coculture with IFNγ-pretreated KCs led to an increased percentage of CD25^+^CD69^+^ T cells in all four T cell subsets (**Figures 1C–F**), and in isolated naïve T cells, CD2 downregulation resulted in a significant reduction in activated naïve T cells (**Figure 1C**). The same was true for the effector T cells (**Figure 1D**). In marked contrast, the activation of effector memory (EM) and central memory (CM) T cells was not significantly influenced by the blockade of CD2-mediated costimulation (**Figures 1E, F**).

Previous studies have shown the need for costimulation during the activation of memory T cells by professional APCs (pAPCs) (24, 25). Therefore, the CD2-independent activation of (memory) T cells by KCs suggests the involvement of other costimulatory receptors that may transmit important costimulatory signals between primary KCs and T cells (memory). However, the role of CD28 could be excluded because previous data from our team and other studies have shown missing surface expression of its ligands, CD80 and CD86, on primary KCs (7, 26).

### CD166 and CD6 accumulate at the contact zone between keratinocytes and PBTs

**Figure 2:**
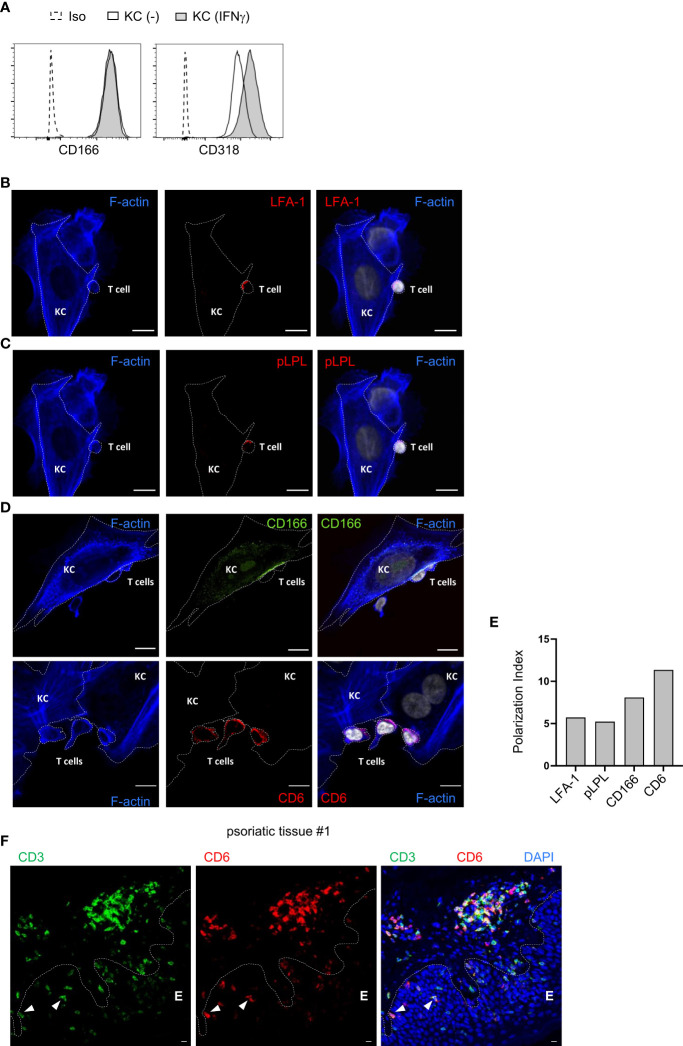
CD166 and CD6 accumulate at the contact zone between primary human keratinocytes and PBTs. **(A)** Flow cytometric analysis of ligands for costimulatory receptors on untreated KCs (black line, white filling) and KCs pretreated with IFNγ for 24 h (black line, grey filling). Isotype control (Iso): dashed line, white filling. **(B–D)** Analysis of the contact zone between T cells and IFNγ-pretreated and SEB-loaded KCs by confocal laser scanning microscopy (40x objective; NA = 1.3) after coculture for 4 h followed by immunofluorescence staining. **(B)** F-actin (blue), LFA-1 (red) and DAPI (white), **(C)** F-actin (blue), phospho L-plastin (pLPL, red) and DAPI (white), (**D**, upper panel) F-actin (blue), CD166 (green) and DAPI (white), (**D**, lower panel) F-actin (blue), CD6 (red) and DAPI (white). Shown are representative pictures for n=4 experiments. Cell borders are marked by dotted lines. Scale bar = 10 µm. Each panel on the right represents the overlay of all three respective stainings. **(E)** Polarization index (PI) of LFA-1, phospho L-plastin (pLPL), CD6 and CD166 at the contact zone of KCs and PBTs. PI: Signal intensity at contact zone/signal intensity on whole cell. **(F)**: Punch biopsies of psoriatic skin were stained for CD3 and CD6. Representative immunofluorescence staining of punch biopsies of skin lesions of psoriasis patient using Opal-4-color IHC kit [CD3 (green), CD6 (red), DAPI (blue)]. Dashed white line represents the border of epidermis **E** to dermis or hair follicle, respectively. White triangles highlight epidermal T cells highly expressing CD6.

The expression of different ligands for costimulatory receptors on untreated and IFNγ-pretreated primary KCs was analyzed by flow cytometry. As described previously, we observed strong expression of CD54 and CD58, whereas CD80 and CD86, the main ligands on pAPCs, were not detected (**Figure S2A**) (7). In addition, CD166 and CD318 were highly expressed in both untreated and IFNγ-pretreated KCs (**Figure 2A**; **Figure S2A**). To further decipher the role of the highly expressed ligands for costimulatory receptors, their localization within the contact zone between IFNγ-pretreated KCs and PBTs was visualized by confocal laser scanning microscopy.

The contact zone between pAPCs and T cells, the immune synapse, is characterized by the accumulation of LFA-1 and pLPL, together with filamentous actin (27). A high accumulation of both proteins was also observed in the contact zone between IFNγ-pretreated KCs and PBTs (**Figures 2B, C**). At T cell/KC contact sites, there was an increased accumulation of CD166 at the KC cell surface (**Figure 2D**, upper panel). The CD166 ligand CD6, which is highly expressed in human T cells (28), was enriched in this contact zone between primary human KCs and PBTs (**Figure 2D**, lower panel).

Together with the high accumulation of LFA-1 and pLPL (**Figure 2E**; **Figure S2B**), these observations indicate that CD166 and CD6 are part of the immune synapse between primary human KCs and T cells. Their interaction is very likely to be involved in the KC-dependent activation of memory T cells.

Immune histological staining of T cells in the epidermis (E) of skin tissue sections derived from psoriasis patients revealed that CD3^+^ T cells highly express CD6 if they were located in close proximity to the border between epidermis and dermis (**Figure 2F**, arrow; **Figure S2C**) while T cells located in the “middle” of the epidermis seemed to downregulate CD6 expression (**Figure 2F**; **Figure S2C**). Overall, these results indicate that the described CD6-mediated costimulation between KCs and T cells can occur within the epidermis of psoriasis patient.

### CD166/CD318-induced costimulatory signals transmitted by CD6 are required for keratinocyte-dependent T cell activation and proliferation

**Figure 3:**
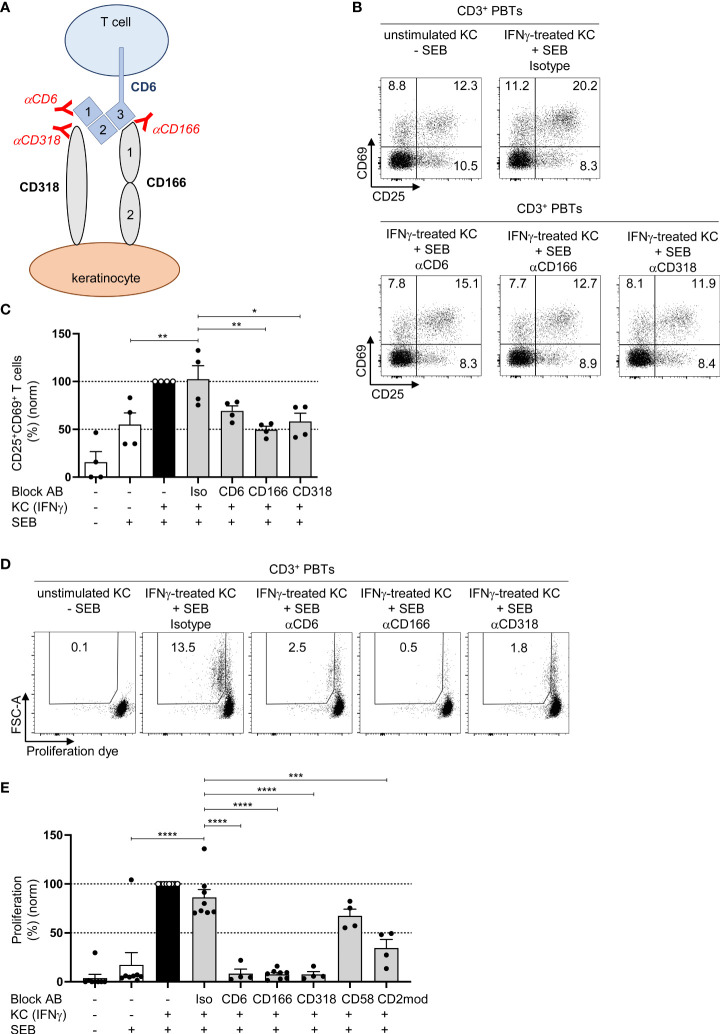
CD166/CD318-induced costimulatory signals transmitted by CD6 are required for keratinocyte-dependent T cell activation and proliferation. **(A)** Graphical scheme of the putative interaction between KCs and T cells and the binding sites of the used blocking antibodies against CD6, CD166 and CD318. αCD6 binds to domain 1 (d1) of CD6, αCD166 binds to domain 1 (d1) of CD166, while αCD318 binds to CD318 which blocks the interaction between CD6 and CD318. **(B, C)** CD3^+^ PBTs were cultured with untreated KCs (white bars) or IFNγ-pretreated KCs (black and grey bars), loaded with SEB (+) and then analyzed for expression of the T cell activation markers CD25 and CD69 (after 24 h) by flow cytometry. **(B)** Representative dot plots and **(C)** statistical evaluation of the effect of isotype control antibodies (iso), or blocking antibodies against CD6, CD166, or CD318 on the CD25 and CD69 expression (n = 4 individual T cell donors). **(D, E)** Effect of isotype control antibodies (iso), or blocking antibodies against CD6, CD166, or CD318 on T cell proliferation after 72 h coculture. Proliferation was assessed by CFDA dilution assay. **(D)** Representative dot plot and **(E)** statistical evaluation (n ≥ 3 individual T cell donors). Data was normalized to PBTs cultured with IFNγ-pretreated KCs loaded with SEB (black bar). Data is represented as mean ± SEM. ****=p<0.0001; ***=p 0.001; **=p<0.01; *=p<0.05.

In addition to its interaction with CD166, CD6 interacts with CD318 (29), which is highly expressed in primary human KCs (**Figure 2A**; **Figure S2A**). We used specific blocking antibodies to elucidate the role of the interaction between CD6 and CD318 or CD166 during the KC-dependent activation of PBTs. As shown in **Figure 3A**, blocking antibodies against CD6 and CD318 inhibited the interaction between CD6 (domain 1) and CD318, whereas anti-CD166 interfered with the binding between CD6 (domain 3) and CD166 (domain 1) (30). The presence of these different blocking antibodies during the coculture of IFNγ-pretreated KCs and PBTs lowered the percentage of activated CD25^+^CD69^+^ T cells to the level observed in the coculture of unstimulated KCs and PBTs (**Figures 3B, C**; **Figures S3A, B**). The inhibitory effect of these blocking antibodies on KC-initiated T cell proliferation was even more prominent. IFNγ-pretreated KCs induced strong proliferation (**Figures 3D, E**, black bar), whereas preventing the interaction between CD6 and CD166 or CD318 resulted in a complete inhibition of PBT proliferation (**Figures 3D, E**, grey bars). Blocking the interaction between CD58 and CD2 using anti-CD58 blocking antibodies showed a slight, though non-significant, reduction in T cell proliferation, while CD2 downregulation caused a significant inhibition of T cell proliferation (**Figure 3E**).

These observations indicate that both CD58/CD2- and CD166/CD318/CD6-mediated costimulation transmit important signals required for KC-induced T cell activation.

### CD6 is not crucial for the adhesion of PBT to KC and IL-2 production

**Figure 4:**
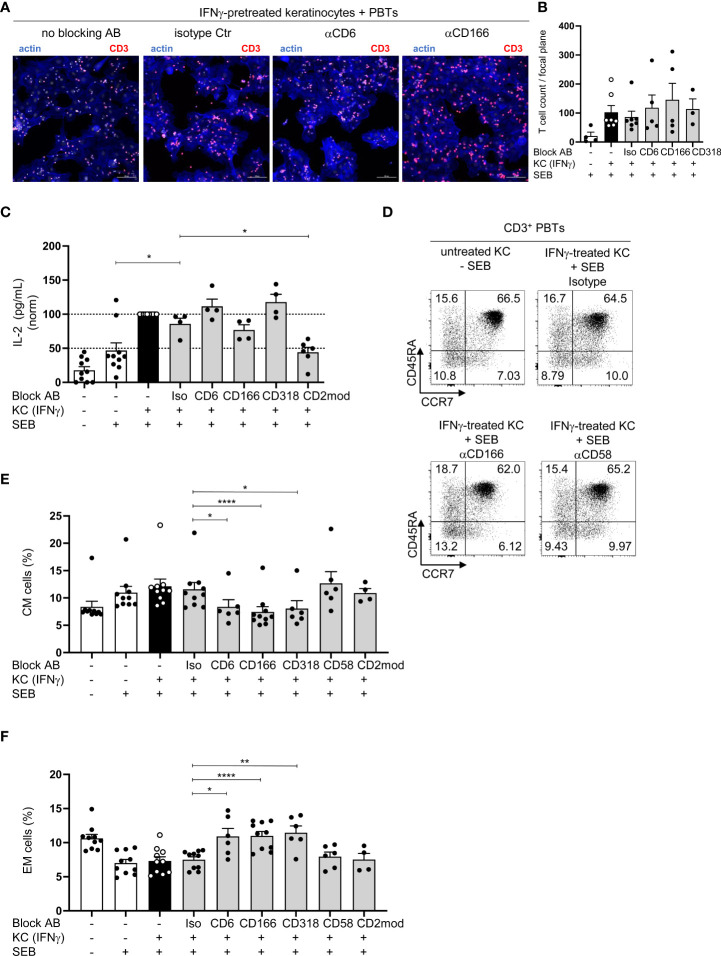
Blockade of CD6-mediated costimulation does not regulate IL-2 production but leads to an increase in EM and a decrease in CM cells. **(A, B)** Adhesion of CD3^+^ PBTs to IFNγ-pretreated KCs (black and grey bars) loaded with SEB in the presence of blocking antibodies (as indicated). Cells were cocultured for 4 h and analyzed by confocal microscopy (20x objective; NA = 0.75). Adhesion was calculated as number of T cells per optical field (n ≥ 3 individual T cell donors). **(A)** Representative immunofluorescence staining for F-actin in KCs and T cells (blue) and CD3 on T cells (red). **(B)** Statistical evaluation of the effect of blocking antibodies against CD6, CD166, or CD318, or isotype control antibodies (Iso) on the T cell adhesion to either untreated KCs (white bar) or IFNγ-pretreated KCs (black bar and grey bars) loaded with (+) SEB. Data is represented as mean ± SEM. **(C–E)** CD3^+^ T cells were cultured for 24 h with untreated KCs (white bars) or IFNγ-pretreated KCs (black and grey bars) loaded with (+) or without **(-)** SEB and then analyzed by flow cytometry. **(C)** Amount of secreted IL-2 after 24 h coculture (n ≥ 4 individual T cell donors). Concentration of secreted cytokines was analyzed by cytokine bead array. *=p<0.05. **(D, E)**: **(D)** Representative dot plots and statistical evaluation of the effect of blocking antibodies or isotype control antibodies after 72 h on percentages of T cell populations **(E)** central memory (CM, CD45RA^-^CCR7^+^) and **(F)** effector memory (EM, CD45RA^-^CCR7^-^) T cells (n ≥ 4 individual T cell donors). Data is represented as mean ± SEM. ****=p<0.0001; **=p<0.01; *=p<0.05.

To elucidate the mode of action of CD6-mediated costimulation during KC-initiated T cell proliferation, the influence of blocking antibodies on key features of T cell activation was analyzed. Since CD166 and CD318 are known adhesion molecules (31–33), the adhesion of PBTs to KCs, which is required for immune synapse formation, was investigated. PBTs showed an enhanced adhesion to IFNγ-pretreated KCs while hardly any binding of PBTs to unstimulated KCs was detected (**Figure S4A**). In contrast to CD54, which was shown to be crucial for adhesion of PBTs to KCs (7), the presence of blocking antibodies against CD6, CD166, or CD318 did not alter the number of PBTs attached to IFNγ-pretreated KCs (**Figures 4A, B**). Thus, although interactions between CD6/CD166 and CD6/CD318 play an important role for KC-induced T cell activation and proliferation, they appear to play a minor role for the adhesion of T cells to KCs.

A pre-requisite for T cell proliferation is the costimulation-dependent production and secretion of interleukin-2 (IL-2). To analyze the concentration of secreted IL-2, the supernatants of the coculture between PBTs and unstimulated or IFNγ-pretreated KCs with or without blocking antibodies were analyzed. As in our previous study (7), IL-2 concentration was significantly higher in the coculture of IFNγ-pretreated KCs and PBTs compared to the coculture with unstimulated KCs (**Figure 4C**, black/white bars). Surprisingly, despite nearly complete inhibition of T cell proliferation, IL-2 production remained unchanged when the interaction between CD6, CD166, and CD318 was blocked. However, IL-2 production was inhibited by the blockade of CD2-dependent costimulation (**Figure 4C**, grey bars). Notably, blockade of CD6/CD166/CD318 or CD2 did not influence the proportion of apoptotic T cells. Approximately 90% of viable T cells were detected after 72 h in the coculture with unstimulated or IFNγ-pretreated KCs, either in the presence or absence of blocking antibodies (**Figure S4B**).

Together, these results indicate a clearly distinguishable role of CD2- and CD6-mediated costimulatory signals during KC-dependent T cell activation.

### Blockade of CD6/CD166/CD318, but not CD2, during coculture of PBT with KC leads to an increase in effector memory and a decrease in central memory T cells

CD45RA and CCR7 expression was analyzed after 72 h to clarify whether the blocked CD6-mediated costimulation caused a shift in the composition of T cell populations, for example, naïve and memory T cells versus effector T cells. The percentages of naïve (CD45RA^+^CCR7^+^) and effector T cell populations (CD45RA^+^CCR7^-^) did not differ between the cocultures containing isotype or blocking antibodies (**Figure 4D**; **Figures S4C–E**). However, the percentage of CM cells (CD45RA^-^CCR7^+^) decreased significantly (**Figures 4D, E**; **Figure S4C**), while EM cells (CD45RA^-^CCR7^-^) in the cocultures increased significantly upon CD6/CD166/CD318 blockade compared with the isotype control antibody (**Figures 4D, F**; **Figure S4C**). Upon blocking CD2 by anti-CD58 or CD2 modulation, no differences were observed in any of the four T cell populations (**Figures 4D–F**; **Figure S4C–E**).

### CD6-mediated phosphorylation of STAT1, STAT3 and STAT5 is important for KC-dependent T cell proliferation

**Figure 5:**
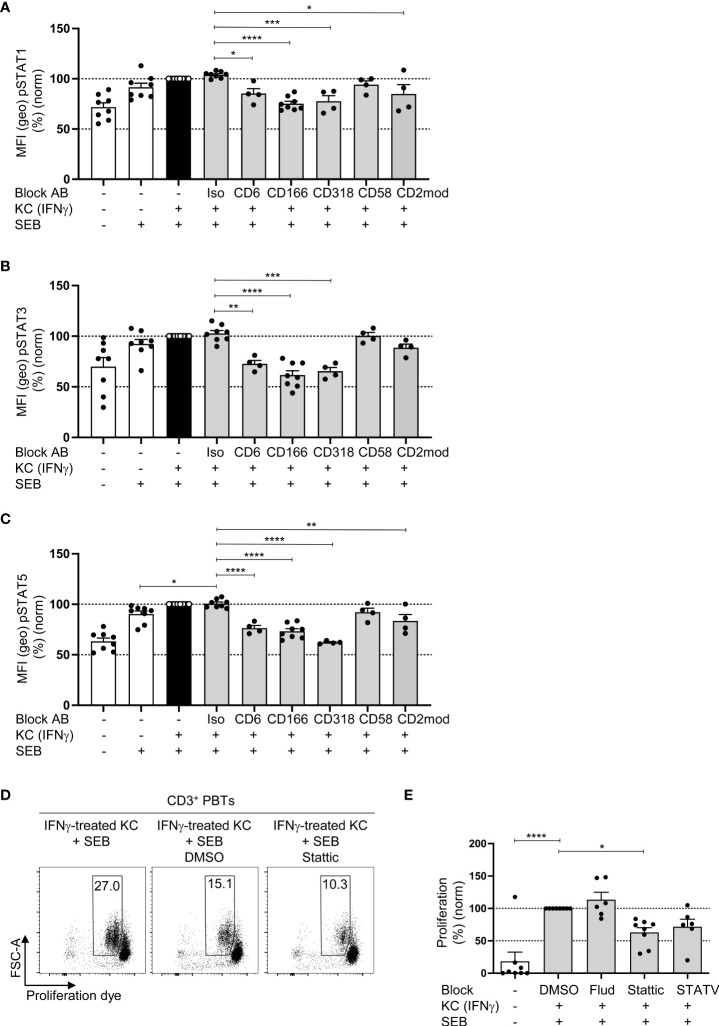
CD6-mediated phosphorylation of STAT1, STAT3 and STAT5 is involved in KC-dependent T cell proliferation. CD3^+^ PBTs were cultured for the indicated time points with untreated KCs (white bars) or IFNγ-pretreated KCs (black and grey bars), loaded with SEB and analyzed by flow cytometry. **(A–C)** Effect of blocking antibodies against CD6, CD166, CD318, CD58, or CD2 downmodulation (CD2mod) and isotype control antibodies (iso) on the phosphorylation of **(A)** STAT1, **(B)** STAT3 and **(C)** STAT5 in PBTs after 24 h coculture (n ≥ 4 individual T cell donors). **(D–E**: **(D)** Representative dot plots and **(E)** statistical evaluation of the effect of fludarabine (Flud), Stattic and STATV treatment on T cell proliferation after 72 h coculture (n ≥ 6 individual T cell donors). Data was normalized to PBTs cultured with IFNγ-pretreated KCs loaded with SEB (black bar). Data is represented as mean ± SEM. ****=p<0.0001; ***=p<0.001; **=p<0.01; *=p<0.05.

To determine whether CD6-dependent costimulation triggers the signal transducer and activator of transcription (STAT) signaling pathways required for T cell proliferation, we quantified the phosphorylation (activation) of STAT1, STAT3, and STAT5 in PBTs cocultured for 24 h with IFNγ-pretreated KCs in the presence or absence of blocking antibodies by flow cytometry. In the coculture with IFNγ-pretreated KCs, the number of phosphorylated STAT1, STAT3, and STAT5 was significantly increased compared with the untreated KCs culture (**Figures 5A–C**). When the interaction between CD6, CD166 and CD318 was blocked, a significant decrease in STAT1, STAT3, and STAT5 phosphorylation was observed.

Analysis of mRNA expression of 27 genes regulated by distinct STAT molecules with the SPRINT system from NanoString Technologies using a customized *Elements code set* revealed that the expression of 18 out of 27 STAT-regulated genes was increased in PBTs cocultured with IFNγ-pretreated KCs compared with PBTs cultured with unstimulated KCs (**Figures S5A, B**). Interestingly, disrupting the interaction between CD6 and CD166 using blocking antibodies caused only a downregulation in the expression of STAT3-regulated genes (FOS and MYC) (**Figure S5C**), whereas CD2 blockade resulted in a diminished expression of genes associated with all three STAT molecules (**Figure S5D**).

To further clarify whether CD6-mediated costimulation triggers T cell proliferation by STAT3-associated pathways, a STAT3 inhibitor (*Stattic*) was used in a 72 h coculture of IFNγ-pretreated KCs and PBTs, followed by the assessment of T cell proliferation (**Figures 5D, E**). *Fludarabine* (STAT1 inhibitor) and *STATV inhibitor* (STAT5 inhibitor) were used as controls. None of the three inhibitors was toxic (**Figure S5E**).

Consistent with the observed correlation between CD6-mediated costimulation and STAT3-associated gene expression, only *Stattic* significantly reduced KC-induced T cell proliferation (**Figures 5D, E**), while *Fludarabine* (STAT1 inhibitor) showed no effect and the STATV inhibitor only a non-significant reduction.

### Blockade of the CD6/CD166/CD318 axis prevents mTOR activation

**Figure 6:**
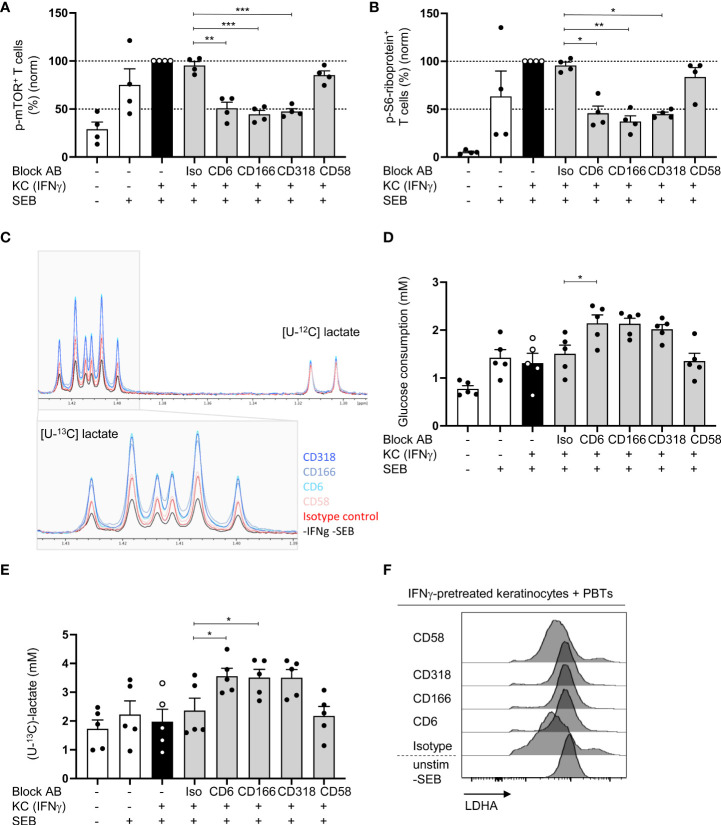
Blocking of CD6/CD166/CD318 interaction prevents mTOR activation and leads to increased aerobic glycolysis. CD3^+^ PBTs were cultured with untreated KCs (white bars) or IFNγ-pretreated KCs (black and grey bars), loaded with SEB and the effects of blocking antibodies against CD6, CD166, CD318 or CD58, or isotype control antibodies (iso) were analyzed. **(A, B)** Flow cytometric analysis of mTOR activation after 72 h coculture. **(A)** Phosphorylation of mTOR at Ser2448, **(B)** phosphorylation of S6-riboprotein (n = 4 individual T cell donors). **(C–E)** Metabolism of isotopically labeled [U-^13^C]-glucose was analyzed in the supernatants of PBTs cultured for 72 h with fixed untreated KCs (white bars) or IFNγ-pretreated KCs (black and grey bars) by ^1^H NMR. **(C)** Representative spectra of [U-^13^C]-lactate. **(D)** Statistical evaluation of glucose consumption and **(E)** concentration of [U-^13^C]-lactate (n = 5 individual T cell donors). Data was normalized to PBTs cultured with IFNγ-pretreated KCs loaded with SEB (black bar). Data is represented as mean ± SEM. ***=p<0.001; **=p<0.01; *=p<0.05. **(F)** Flow cytometry of intracellular LDHA expression in PBTs cocultured for 72 h with untreated or IFNγ-pretreated KCs either in the presence or absence of blocking antibodies against CD6, CD166, CD318 or CD58 or isotype control antibodies. Shown are representative histograms of one out of 6 independent experiments.

We analyzed the influence of CD6-mediated costimulation on STAT-regulated processes to further elucidate the link between CD6-dependent T cell costimulation, STAT signaling, and T cell proliferation. STAT signaling plays a crucial role in regulating T cell metabolism (34, 35). Consequently, we investigated the influence of blocking the CD6/CD166/CD318 axis on mTOR, the key coordinator of metabolic processes involved in cell growth and proliferation (36). mTOR is activated *via* phosphorylation at Ser2448. Upon coculture with IFNγ-pretreated KCs, a strong phosphorylation of mTOR at Ser2448 in T cells was observed. Compared with the coculture with isotype control antibodies, this phosphorylation was significantly inhibited when CD6-mediated costimulation was blocked [**Figure 6A** (% positive cells), **Figure S6A** (MFI geo)]. In contrast, blockade of CD2/CD58 did not influence mTOR phosphorylation.

Next, the presence of phosphorylated S6-ribosomal protein (phospho-S6-riboprotein), which is regulated by mTOR (37), was assessed. In line with the data on mTOR phosphorylation, coculture with IFNγ-pretreated KCs led to increased amounts of phospho-S6-riboprotein in PBTs. This increase was significantly reduced upon blockade of the CD6/CD166/CD318 interaction [**Figure 6B** (% positive cells), **Figure S6B** (MFI geo)]. Importantly, S6-riboprotein phosphorylation was not influenced by the blockade of CD2 stimulation. These data suggest a pivotal role of CD6-mediated costimulation in regulating the mTOR pathway and its associated metabolic processes in human T cells.

### Blockade of the CD6/CD166/CD318 axis leads to increased aerobic glycolysis

To address the involvement of CD6-mediated costimulation in T cell metabolic processes, we first analyzed its effect on aerobic glycolysis. The production of metabolic intermediates was quantified by ^1^H-nuclear magnetic resonance (^1^H-NMR). To achieve this, PBTs were cultured with untreated or IFNγ-pretreated KCs in the presence of [U-^13^C]-glucose. After 24 and 72 h, the amount of consumed glucose and the concentration of the produced [U-^13^C]-lactate was quantified in the coculture supernatant by ^1^H-NMR. Significantly enhanced [U-^13^C]-glucose consumption was detected after 72 h (**Figure S6C**). The same observation was made regarding [U-^13^C]-lactate concentration. After 72 h, the [U-^13^C]-lactate concentration shifted from 1.5 mM to more than 2 mM in the coculture of PBTs with untreated KCs without SEB and IFNγ-pretreated KCs loaded with SEB (**Figure S6D**). Thus, approximately 75% of the consumed [U-^13^C]-glucose was converted to [U-^13^C]-lactate (1.5 mM glucose ➔ 2.2 mM lactate).

Since the highest [U-^13^C]-glucose consumption, [U-^13^C]-lactate production, and glycolytic rate were observed after 72 h, this time point was used for the coculture with blocking antibodies and subsequent ^1^H-NMR analysis (**Figure 6C**). In parallel, T cell proliferation was assessed after 72 h (**Figure S6E**). All antibodies inhibited T cell proliferation (**Figure S6E**). However, surprisingly, glucose consumption was even higher in the presence of blocking antibodies against CD6, CD166, and CD318, whereas CD2 blockade by anti-CD58 showed no difference (**Figure 6D**). The same effect was observed when the [U-^13^C]-lactate concentration was analyzed. After blocking CD6, CD166, or CD318, the [U-^13^C]-lactate concentration increased to 3.56 mM, whereas CD58 blocking had no effect (**Figures 6C, E**).

To understand the mechanism behind the enhanced *de novo* lactate production upon blockade of CD6-mediated costimulation, we analyzed the protein levels of the key enzyme lactate dehydrogenase A (LDHA) regulating this step of (aerobic) glycolysis (38). Similar to T cells stimulated by professional APCs (39), LDHA expression in PBTs was significantly downregulated upon stimulation with IFNγ-pretreated KCs compared with unstimulated PBTs or the coculture of PBTs and untreated KCs (**Figure 6F**; **Figure S6F**). Interestingly, the downregulation of LDHA expression was prevented by the blockade of CD6, CD166, and CD318 interaction, whereas CD58 blockade had no effect on LDHA expression (**Figure 6F**; **Figure S6F**). Thus, CD6-mediated costimulation appears to suppress LDHA expression. This may result in an increased shuttling of pyruvate into the TCA cycle, leading to the production of precursors required for nucleotide, lipid, and protein synthesis.

### Blockade of the interaction between CD6, CD166 or CD318 lowers the mitochondrial membrane potential and increases levels of reactive oxygen species

**Figure 7:**
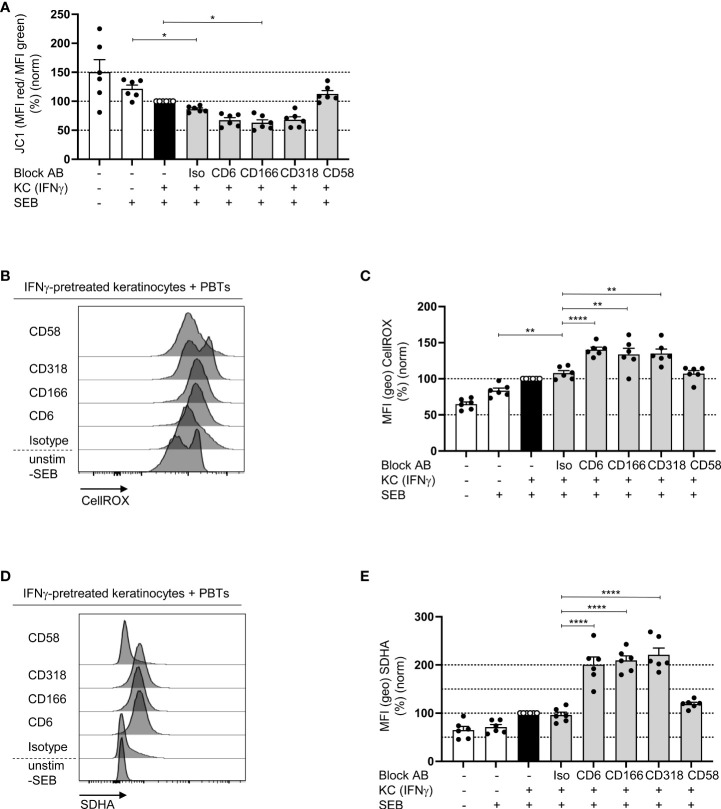
Blockade of the interaction between CD6, CD166 or CD318 lowers the mitochondrial membrane potential and increases ROS levels. CD3^+^ PBTs were cultured for 72 h with untreated KCs (white bars) or IFNγ-pretreated KCs (black and grey bars), loaded with SEB and the effects of blocking antibodies against CD6, CD166, CD318 or CD58, or isotype control antibodies (iso) were analyzed. **(A)** Mitochondrial membrane potential assessed by JC-1 (ratio between aggregates and monomers) after 72 h coculture (n = 6 individual T cell donors). **(B, C)** Representative histograms **(B)** and statistical evaluation **(C)** of intracellular ROS (assessed by CellROX) in PBTs (n = 6 individual T cell donors). **(D, E)** Intracellular SDHA expression in PBTs. **(D)** Representative histograms and **(E)** statistical evaluation (n = 6 individual T cell donors). Data was normalized to PBTs cultured with IFNγ-pretreated KCs loaded with SEB (black bar). Data is represented as mean ± SEM. ****=p<0.0001; **=p<0.01; *=p<0.05.

While naïve T cells predominantly use OXPHOS as the main energy source, proliferating T cells switch to aerobic glycolysis, which is associated with lower mitochondrial membrane potential (21). To determine whether CD6-dependent costimulation regulates OXPHOS, which is localized in the mitochondria, first, the mitochondrial membrane potential (ΔΨM) was analyzed using the membrane-permeable fluorescent dye JC-1. In PBTs cultured with IFNγ-pretreated KCs, ΔΨM was significantly reduced compared with that in PBTs cultured with untreated KCs (**Figure 7A**). ΔΨM was even lower upon blockade of CD6-dependent costimulation, while it remained unchanged between the isotype control and CD58 blockade (**Figure 7A**).

The production of reactive oxygen species (ROS) is another function of mitochondria during T cell activation and proliferation. At low concentrations, ROS enhances proximal TCR signaling (40). However, high amounts of ROS can induce oxidative stress, which plays a central role in inducing T cell hyporesponsiveness. Upon coculture of PBTs and IFNγ-pretreated KCs ROS production was significantly enhanced compared to the coculture with untreated KCs (**Figures 7B, C**), which is in line with previous data obtained with T cells activated by pAPCs (40, 41). Interestingly, upon blocking the interaction between CD6, CD166, and CD318, ROS production increased even further; whereas, on CD58 blockade, the level of ROS was comparable to that of the isotype control (**Figures 7B, C**).

To understand the link between blocked CD6-dependent costimulation, reduced ΔΨM, and increased ROS production, the expression of key metabolic enzymes was analyzed. Since recent publications have shown an important role for mitochondrial complex II during ROS production (42, 43), we analyzed the expression of its largest subunit, succinate dehydrogenase A (SDHA). Corresponding with the elevated ROS levels, SDHA expression was significantly upregulated in PBTs cocultured with IFNγ-pretreated KCs in the presence of the blocking antibodies against CD6, CD166, and CD318 compared with the coculture with isotype controls (**Figures 7D, E**). Blocking CD2 with anti-CD58 had no effect.

Together, these data indicate that costimulation through CD6, but not CD2, may promote OXPHOS and at the same time significantly downregulate SDHA expression, thereby preventing excessive ROS production in human T cells.

### Blockade of the interaction between CD6, CD166 or CD318 impairs fatty acid synthesis but not uptake

Alterations in mitochondrial function may influence not only ROS production and energy balance but also the synthesis of important building blocks required for T cell proliferation. One of these building blocks is FAs, the major components of cell membranes synthesized by two systems, one of which resides in the cytoplasm and the other in the mitochondria (44).

**Figure 8:**
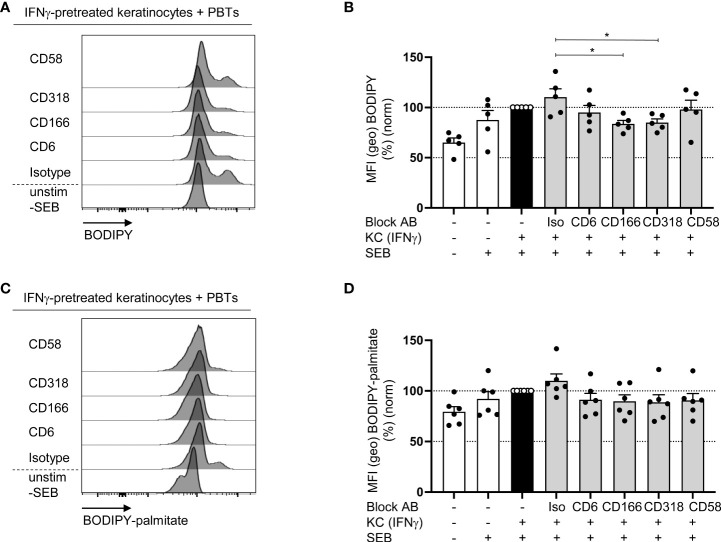
Blockade of the interaction between CD6, CD166 or CD318 impairs fatty acid synthesis **(A, B)** but not fatty acid uptake **(C, D)**. CD3^+^ PBTs were cultured for 72 h with untreated KCs (white bars) or IFNγ-pretreated KCs (black and grey bars), loaded with SEB and the effects of blocking antibodies against CD6, CD166, CD318 or CD58 or isotype control antibodies (iso) were analyzed. **(A, B)** Representative histograms **(A)** and statistical evaluation **(B)** of BODIPY staining in PBTs analyzed by flow cytometry (n = 5 individual T cell donors). **(C, D)** Representative histograms **(C)** and statistical evaluation **(D)** of BODIPY-palmitate staining in PBTs analyzed by flow cytometry (n = 6 individual T cell donors). Data was normalized to PBTs cultured with IFNγ-pretreated KCs loaded with SEB (black bar). Data is represented as mean ± SEM. *=p<0.05.

To analyze whether CD6-mediated costimulation is involved in the regulation of FA synthesis, the content of neutral lipids in PBTs was determined using BODIPY493/503. PBTs cocultured with IFNγ-pretreated KCs showed a higher non-polar lipids content than the culture with untreated KCs (**Figures 8A, B**). Consistent with our previous data showing impaired T cell proliferation (**Figure 3E**), blocked interactions between CD6, CD166, or CD318 resulted in significantly diminished BODIPY493/503 fluorescence (**Figures 8A, B**). BODIPY493/503 stains all neutral lipids within a cell and does not discriminate between newly synthesized FAs and FAs taken up by the extracellular milieu. Therefore, the uptake of FAs in PBTs cocultured with IFNγ-pretreated KCs was determined by BODPY-FL-C_16_ (palmitate bound to BODIPY). Here, no significant changes were detected upon blockade of the interaction between CD6, CD166, or CD318 compared with the isotype control (**Figures 8C, D**). Thus, CD6-mediated costimulation appears to primarily support the synthesis but not uptake of FAs.

## Discussion

The regulation of T cell metabolism has been mainly attributed to costimulation through CD28. In this study, we demonstrated a crucial role of CD6 in regulating metabolic processes upon T cell activation in the absence of CD28-mediated costimulation. Recently, we showed that primary human KCs, which do not express CD80 or CD86 (the ligands of CD28), can activate primary human T cells *via* costimulation through CD2 (7). Here we found that, in addition to CD2 (7), CD6 plays a crucial role in KC-induced costimulation and the induction of T cell proliferation. Interestingly, costimulation through CD6, but not CD2, regulates T cell metabolism upon KC-mediated activation of human T cells. Costimulation *via* CD6 activates the mTOR pathway and downregulates the expression of LDHA and associated lactate production. Moreover, CD6-mediated signals enhance mitochondrial processes that promote OXPHOS and FA synthesis, and diminish ROS production. At the same time, CD6-mediated costimulation counteracts the metabolic switch of T cells to aerobic glycolysis. Therefore, a unique role of CD6-mediated costimulation during KC-induced activation and subsequent proliferation of peripheral T cells was identified.

Our data provide evidence that CD6 accumulates at the immunological synapse and activates intrinsic signaling pathways. STAT signaling seemed to be involved in these processes since interrupted CD6-dependent costimulation resulted in significantly reduced phosphorylation of STAT1, STAT3, and STAT5. Gene expression analysis as well as the use of different STAT inhibitors indicated that, of these signalling molecules, STAT-3 appears to play a major role for the regulation of T cell proliferation following KC-mediated costimulation through CD6.

In addition, mTOR-dependent pathways are upregulated by CD6-mediated signaling, which initiates processes required to fuel the metabolic needs of proliferating cells. Accordingly, upon blocking CD6/CD166/CD318 interactions, the key metabolic regulator mTOR showed reduced activity, which most likely caused the downregulation of mitochondrial function. An impaired energy supply through OXPHOS upon blockade of CD6-mediated costimulation was supported by reduced ΔΨM, as measured by JC-1 staining, and upregulated intracellular ROS levels. Although increased ROS production is an early feature of T cell activation to support TCR-signaling (40), extensive ROS generation has been linked to diminished electron transport chain activity and reduced antioxidative capacity of the mitochondria (45). The observed upregulation of ROS production in the absence of CD6-dependent costimulation could be explained by the dramatically enhanced SDHA expression since SDHA participates in the respiratory chain, required for ATP synthesis, and in the TCA cycle by converting succinate to fumarate (46).

Together with the diminished ΔΨM, it seems that the blockade of CD6-mediated costimulation inhibits processes involved in the respiratory chain, while the TCA remains functional, which would allow the production of essential building blocks, such as amino acids, FAs, or nucleic acids, required for T cell activation. However, it remains unclear whether SDHA expression is directly regulated by CD6-mediated costimulation.

Excessive ROS production upon blockade of CD6-mediated costimulation may in turn suppress the activity of mTOR and other transcription factors, such as NFAT, leading to a misbalanced anabolic pathway failing to fuel the metabolic requirements and compromising T cell proliferation upon activation by KCs (47, 48). Moreover, in a pro-oxidative milieu, oxidation of key regulators of the actin cytoskeleton, namely cofilin and L-plastin, leads to T cell hyporesponsiveness or even T cell death (27, 49, 50).

Proliferating cells usually shift their metabolic pathways towards aerobic glycolysis to produce biosynthetic precursor molecules required for cell division. This metabolic reprogramming is tightly regulated by mTOR activation (51, 52). However, glucose consumption, LDHA expression, and lactate production increased upon the blockade of CD6, CD166, or CD318. Thus, costimulation through CD6 limits the classical metabolic switch in T cells. Most likely, T cells that received the first stimulus through TCR but no second signal through CD6 turned into (metabolic) stress, which they try to counterbalance by switching to aerobic glycolysis (53). Furthermore, cells use enhanced glycolysis to cope with oxidative stress. Elevated glycolysis promotes the pentose phosphate pathway, which, in turn, leads to increased levels of NADPH, which binds to excessive ROS.

High OXPHOS and low aerobic glycolysis are also features of TRM and CM T cells, whereas the opposite holds true for EM T cells (54). In this context, blockade of CD6 and CD166/CD318 upon KC-induced T cell activation decreased the number of CM T cells and increased the number of EM T cells. This could explain the observed elevated levels of aerobic glycolysis upon CD6/CD166/CD318 blockade. Since CM T cells have been shown to be the most effective precursors of human skin TRM (55), CD6-mediated costimulation may be crucial for the homeostasis of skin TRMs.

The link between disrupted CD6-mediated costimulation and inhibited proliferation could be explained by oxidative stress through high ROS production either in addition to, or instead of influences on key signaling events. Thus, CD6 is strongly associated with the TCR/CD3 complex (56) and functions as a signalosome during TCR signaling. CD166 binds to domain 3 of CD6, whereas CD318 binds to domain 1, leading to the translocation of these complexes to the center of the immune synapse of professional APCs and T cells (29, 57). Following T cell costimulation, SLP-76, Vav1, and other adaptor and signaling proteins interact with the phosphorylated cytoplasmic domain of CD6, leading to the activation of MAP kinase pathways, which finally activates mTOR complex 1 (58, 59). Within the immune synapse of APCs and T cells, the linker for the activation of T cells (LAT) acts as another signaling hub crucial for the diversification of TCR signals. Through selective depletion of LAT or CD6, Mori et al. demonstrated that CD28-mediated costimulatory signals are mainly transmitted by the transmembrane protein LAT while CD28-independent signals are transmitted by the CD6 signalosome (60). This observation is consistent with the essential role of CD6 during KC-induced T cell proliferation, as T cells activated by primary human KCs do not receive signals transmitted *via* CD28.

In the blocking experiments, we used a CD6 antibody which binds to domain 1 of CD6 and blocks the interaction between CD6 and CD318. Furthermore, we used an antibody against CD166 which blocks the interaction between CD166 and domain 3 of CD6. It seems that both, the interaction of CD6D1 with CD166 and CD6D3 with CD318, transmit signals essential for KC-induced T cell activation, which cannot be compensated by each other since the blockade of only one of these causes abolished T cell proliferation. Although diverse modes of action of different CD6 blocking antibodies against domain 1 or 3 have been described (61–63), it remains unclear how CD318 or CD166 signal through CD6.

The 2D coculture system used in this study focuses on the direct interaction between primary human KCs and T cells, which was also observed in the epidermis of skin lesions derived from psoriasis patients. In order to take other cellular interactions into account and to resemble skin morphology in our experiments as close as possible, a human skin equivalent model and human skin explant models that also include other cell types such as fibroblasts, will be used for future experiments.

The essential role of CD6-dependent costimulation complements our previous study showing the crucial role of CD2-dependent costimulation during KC-mediated activation of naïve T cells and subsequent Th1 differentiation (7). Thus, in the absence of natural ligands for CD28, other costimulatory receptors, such as CD2 or CD6, take over to control essential processes during the KC-mediated activation of T cells. While costimulation through CD2 is important to activate naïve T cells and primarily induces the production of IL-2 or IFNγ as well as differentiation into Th1 cells, CD6-dependent costimulation plays an essential role during the KC-dependent activation of already differentiated memory T cells. During this process, signals transmitted through CD6 and CD166/CD318, but not CD2, regulate the metabolic processes required for efficient T cell activation and proliferation. This observation reveals a mechanistic explanation for how the availability of different ligands for costimulatory receptors dictates the fate of T cells. The present study focused on PBTs consisting of both, CD4^+^ and CD8^+^ T cells. Further studies should address the point, whether these distinct T cell subpopulations may rely on different costimulatory signals as well.

Since most epidermal T cells do not express CD28 but express CD2 and CD6 (7), keratinocyte-induced costimulation through CD2 and CD6 could be a potential mechanism by which skin-homing or skin resident T cells can be activated/sensitized under proinflammatory conditions. Accordingly, topical blockade of CD6/CD166/CD318 or CD2/CD58 in the skin may provide novel options to selectively influence different T cell populations.

Several clinical studies have demonstrated the efficacy of itolizumab, a humanized monoclonal antibody that selectively targets CD6, in the systemic treatment of moderate-to-severe psoriasis (64–66). Itolizumab binds to domain 1 of CD6 and inhibits T cell activation, proliferation, and secretion of proinflammatory cytokines involved in the pathogenesis of psoriasis (62). By demonstrating the pivotal role of CD6 in the CD28-independent activation of primary human T cells by KCs, our data suggest that the efficacy of itolizumab in the treatment of inflammatory skin pathologies may, at least in part, be due to a metabolic switch in T cells and the inhibition of mTOR signal transmission.

Furthermore, our coculture setup, especially the use of alloreactive T cells, mimics the pathophysiology of GvHD. Previous studies have reported the therapeutic benefit of itolizumab in patients with acute GvHD (67) which was investigated in a phase I/II study (NCT03763318). Thus, our data provide another line of evidence and molecular explanation for the potential beneficial use of itolizumab or other CD6-targeting inhibitors in the treatment of patients with GvHD. Alternative immunotherapeutic approaches based on single-domain antibodies or nanobodies targeting the interaction between CD6/CD166 and CD318 could be even more effective. In contrast to complete monoclonal antibodies, these smaller molecules should be more efficient in entering the immune synapse and disrupting specific interactions in already formed KC-T cell contacts (68, 69). This will be the subject of further investigations.

Overall, our current study revealed the fundamental role of the interaction between CD6 and its ligands CD166 and CD318 during the activation of T cells through KCs in the absence of CD28. Furthermore, our findings provide clear evidence that CD6, in addition to its known function as a signalosome, can regulate metabolic pathways essential for T cell proliferation and central memory T cell formation. Importantly, given the lack of CD86 and CD80 expression in KCs, our study indicates new therapeutic options for inflammatory skin diseases, targeting CD6 directly in the skin rather than using systemic routes. This approach may minimize the side effects of CD6 targeting.

## Materials and methods

### Primary cells and cell lines

Primary normal human epidermal keratinocytes (NHEK; named primary human KCs in the manuscript) of juvenile foreskin from pooled donors were purchased from Promocell. KCs were cultured in serum-free medium (Keratinocyte growth medium 2 (KGM-2), Promocell, Heidelberg). In some experiments, KCs were treated overnight with 100 ng/mL IFNγ (Biolegend, San Diego, California) (as indicated in the respective figures).

Peripheral blood mononuclear cells (PBMCs) were obtained from heparinized blood of healthy donors using Ficoll-Hypaque (Linaris, Dossenheim) density-gradient centrifugation. PBTs were isolated from PBMCs using the Pan T cell isolation kit (Miltenyi Biotec, Bergisch Gladbach). This study was approved by the Ethics Committee of Heidelberg University (CRC156/B04).

### Keratinocyte-T cell coculture and analysis of T cell activation marker expression

KCs, either untreated or incubated with 100 ng/mL IFNγ overnight, were cultured with 5 µg/mL *S. aureus*-derived enterotoxin B (SEB) (Sigma Aldrich, St. Louis, Missouri) for 1 h at 37 °C, 5 % CO_2_. KCs were washed three times with KGM-2 before PBTs were added in serum-free medium (XVIVO-15, Lonza, Basel). The cocultures were incubated for 24 h at 37°C, 5 % CO_2_ followed by the analysis of the surface expression of activation markers CD25 and CD69 by flow cytometry.

### T cell proliferation assay

To assess T cell proliferation, PBTs were stained with 1 µM 5-Carboxyfluorescein Diacetate (CFDA) (Thermo Fischer Scientific, Waltham, Massachusetts) for 20 min at 37 °C, 5 % CO_2_ before they were cultured in serum-free medium (XVIVO-15, Lonza, Basel) with KCs. The cocultures were incubated for 72 h at 37 °C, 5 % CO_2_ and T cell proliferation was analyzed by destaining of CFDA using flow cytometry.

### Cytokine secretion assay

To assess the quantity of secreted cytokines, the supernatant of the coculture between PBTs and KCs was collected and 13 cytokines were analyzed by LEGENDplex Human Th Panel (Biolegend, San Diego, California) according to manufacturer’s protocol.

### Phospho-flow cytometry

For staining of phosphorylated proteins and metabolic enzymes or transcription factors, cells were fixed with Cytofix Fixation buffer (BD Bioscience, Franklin Lakes, New Jersey) for 10 min at 37 °C, 5 % CO_2_. T cells were permeabilized with Phosflow Perm Buffer III (BD Bioscience, Franklin Lakes, New Jersey) for 30 min on ice, before cells were stained with antibodies against surface markers and phospho-specific antibodies [p_Ser727_STAT1 (A15158B) (Biolegend, San Diego, California), p_Tyr705_STAT3 (4/P-STAT3) (BD Bioscience, Franklin Lakes, New Jersey), p_Tyr694_STAT5 (47/Stat5) (BD Bioscience, Franklin Lakes, New Jersey), p_Ser2448_mTOR (MRRBY) (Thermo Fisher Scientific, Waltham, Massachusetts), p_Ser235/236_S6 Ribosomal Protein (D57.2.2E) (Cell Signaling Technology, Danvers, Massachusetts), LDHA (C4B5) (Cell Signaling Technology, Danvers, Massachusetts) or SDHA (D6J9M) (Cell Signaling Technology, Danvers, Massachusetts)].

### Phenotyping of keratinocytes

Adherent KCs, either untreated or incubated with 100 ng/mL IFNγ (Biolegend, San Diego, California) overnight, were detached with warm trypsin/EDTA (Gibco, Thermo Fisher Scientific, Waltham, Massachusetts). The reaction was stopped by adding RPMI1640 + 10 % FBS. Surface expression of ligands for costimulatory receptors (CD54, CD58, CD80, CD86, CD166 and CD318) was analyzed by flow cytometry. Used antibodies are listed below.

### Antibody-mediated blocking of costimulatory molecules

In some coculture experiments, blocking antibodies (final concentration: 10 µg/mL) against CD6 (M-T605) (BD Bioscience, Franklin Lakes, New Jersey), CD166 (3A6) (Biolegend, San Diego, California), CD318 (CUB1) (Biolegend, San Diego, California), CD58 (TS2/9) (Biolegend, San Diego, California) and isotype control (T8E5) (*In vivo* Gen, San Diego, California) were added to KCs together with PBTs in serum-free medium (XVIVO-15, Lonza, Basel). T cell activation was assessed after 24 h by analyzing the expression of activation markers CD25 and CD69. T cell adhesion was assessed after 24h, by staining with CD3-APC antibody. Adherent T cells were counted by immune fluorescence microscopy. T cell proliferation was analyzed by destaining of CFDA using flow cytometry after 72 h.

### CD2-downmodulation on PBTs

PBTs were incubated with anti-human CD2 (IgM, Clone 2S5AE4; final concentration 0.04 mg/mL) overnight at 37 °C, 5 % CO_2_ in RPMI1640 + 10 % FBS.

### STAT inhibitor-treatment of PBTs

PBTs were incubated with 1 µM Fludarabine (Tocris, Bristol), 1 µM Stattic (Cayman chemical, Ann Abor, Michigan), 10 µM STATV inhibitor (Cayman chemical, Ann Abor, Michigan) or 0.1% DMSO (Sigma Aldrich, St. Louis, Missouri) for 1 h at 37 °C, 5 % CO_2_ in serum-free medium (XVIVO-15, Lonza, Basel). These cells were washed once in serum-free medium (XVIVO-15, Lonza, Basel), before they were added to IFNγ-pretreated and SEB-loaded primary KCs and were cocultured at 37 °C, 5 % CO_2_. T cell proliferation was analyzed by destaining of CFDA using flow cytometry after 72 h.

### Quality control and quantitation of RNA

Absorbance spectra and fluorometric quantity assessment of total RNA were determined by the DeNovix spectrophoto- and fluorometer (DeNovix Inc., Wilmington, DE, USA) and the Qubit fluorometer (ThermoFisher Scientific). RNA quality was assessed on the Agilent 2100 Bioanalyzer (Agilent Technologies, Inc, Santa Clara, CA, USA). Samples were stored at – 80°C until further processing.

### nCounter target gene expression analysis

PBTs were cultured for 4 h with either unstimulated or IFNγ-pretreated KCs in the presence or absence of blocking antibodies or STAT inhibitors. Total RNA was then extracted from PBTs using Direct-zol RNA Miniprep Kit (Zymo Research). All RNA samples were quantified using Qubit RNA assay kit and RNA integrity was assessed using Agilent 2100 Bioanalyzer system.

nCounter target gene expression analyses were carried out using the SPRINT system from nanoString Technologies (Seattle, WA, USA). The nCounter technology allows multiplexed gene expression analysis based on simultaneous hybridization and digital quantification of fluorescently-labelled oligonucleotide probes (70). In short, a reporter probe which carries a fluorescent barcode, a biotinylated capture probe that immobilizes the complex for data collection and two target gene-specific oligonucleotide probes A+B specifically hybridize to each target transcript. For the investigation of respective expression profiles, a customized *Elements codeset*, composed of 27 target genes and 7 reference genes (see **Table S1**), was applied. After hybridization, excess probes and tags as well as unbound non-target nucleic acids are washed away in a purification step. Purified reporter/capture probe: target nucleic acid complexes are then applied on a streptavidin-coated imaging surface and immobilized *via* the biotinylated capture probe. Immobilized reporters are aligned, stretched, and immobilized again in order to align parallel fluorescent barcodes for imaging. Afterwards the cartridge is scanned, and the ordered fluorescent segments on the attached reporter probe define each target molecule of interest. The relative expression level of each target corresponds to the code count number of fluorescent barcodes for each target, termed ‘codeset counts’.

Evaluation of data, background correction and normalization were carried out using the nSolver Analysis Software (version 4.0) provided by nanoString Technologies (“https://www.nanostring.com/products/analysis-software/nsolver”).

### Immunofluorescence microscopy

KCs, either untreated or incubated with 100 ng/mL IFNγ overnight, were cultured on coverslips. These KCs were loaded with SEB for 1 h at 37 °C, 5 % CO_2_ and washed three times with serum-free KGM-2 before PBTs were added in serum-free medium (XVIVO-15, Lonza, Basel). After coincubation for different time periods (as indicated in the figures), cells were fixed with 1.5 % PFA for 20 min, permeabilized with 0.1 % saponin (in PBS + 10 % FBS) and stained with DAPI (Sigma Aldrich) (100 ng/mL), phalloidin-AF488 (Thermo Fisher Scientific, Waltham, Massachusetts) (0.4 U/mL), anti-phospho-L-plastin (UHZ) (Epitomics, Abcam, Cambridge, UK) (1 µg/mL) or anti-CD11a (LFA-1; Hl111) (Biolegend, San Diego, California) (2 µg/mL) anti-CD6 (M-T605) (BD Bioscience, Franklin Lakes, New Jersey) or anti- CD166 (3A6) (Biolegend, San Diego, California).

Anti-rb-Cy3 (Dianova, Hamburg) (1 µg/mL) was used as secondary antibody. Laser scanning confocal microscopy was performed using a Nikon A1R (20x objective; NA = 0.75; 40x objective; NA = 1.3).

The polarization index (PI) of LFA-1, phospho-L-plastin, CD6 and CD166 was calculated by dividing the signal intensity within the contact zone by the signal intensity on the whole cell. The signal intensity was normalized to the area of the respective region of interest (ROI).

### Immunofluorescence staining of human skin tissue

Included patients for immunohistochemistry (IHC) analysis: skin biopsies (5mm punch biopsy) were collected from patients with plaque psoriasis. Skin was also taken from healthy volunteers. This study was approved by the Ethics Committee of Heidelberg University (S-392/2010).

Fluorescence staining of human skin tissue (5 mm biopsies) was performed using the Opal 4-Color Manual IHC Kit (PerkinElmer, Waltham, Massachusetts). In brief, formalin-fixed paraffin-embedded tissue sections (2 µm) were deparaffinized with series of xylenes, and rehydrated with decreasing concentrations of alcohol followed by formaldehyde fixation. The slides were subjected to antigen retrieval using microwave treatment followed by cooling at room temperature. Tissue sections were blocked with Antibody Diluent/Block reagent (PerkinElmer, Waltham, Massachusetts) and incubated for 1 h with primary antibody against CD3 (SP7, DCS Innovative Diagnostic-System, Hamburg, Dilution 1:200) in a humidified chamber. Opal polymer HRP Ms + Rb (PerkinElmer, Waltham, Massachusetts) was used as secondary antibody, followed by Opal signal generation using Opal 520 Fluorophore (PerkinElmer, Waltham, Massachusetts). Slides were placed again in antigen retrieval solution and heated using microwave treatment to strip the primary-secondary-HRP complex. This procedure was repeated with primary antibodies against CD6 (M-T605) (BD Bioscience, Franklin Lakes, New Jersey) (Opal 570 Fluorophore). Nuclei were finally stained using Roti-Mount FluorCare DAPI (Roth, Karlsruhe). As negative control, immunofluorescence staining without primary antibodies was used.

### NMR analysis of media

PBTs were cultured with KCs for different time periods (as indicated in the figures) in cell culture medium containing 5mM of [U-^13^C]-glucose. At the specified time points, coculture supernatants were collected and analyzed by ^1^H NMR spectrometry.

^1^H NMR spectra of cell-culture media were acquired using a 600 MHz Bruker spectrometer equipped with a 5-mm indirect detection probe, as previously described (71). Sodium fumarate (10 mM), dissolved in a 0.2 M phosphate buffer solution prepared with D_2_O (99.9%), was added as an internal standard to enable metabolite quantification. Samples consisting of 140 μL of culture media plus 35 μL of fumarate standard were analyzed. Metabolism of isotopically labeled [U-^13^C]-glucose contained in the culture medium gives rise to ^1^H-^13^C satellites of lactate. The resonances are well resolved in the spectrum and allow concentration determinations necessary for evaluating glycolysis and oxidative metabolism. Metabolite levels were measured by ^1^H NMR spectra deconvolution using the line-fitting sub-routine of TopSpin v4.0.9 NMR software (Bruker, Karlsruhe, Germany).

### T cell metabolism and intracellular ROS content

PBTs were cultured with KCs for different time periods (as indicated in the figures). At the specified time points, PBTs were collected and analyzed for different metabolic processes.

JC-1 (ThermoFisher Scientific, Waltham, Massachusetts) was used to assess changes in the mitochondrial membrane potential. When the mitochondrial membrane is not polarized JC-1 stays as a monomer emitting fluorescence in the green-area of the light spectrum, while when the membrane is polarized JC-1 builds aggregates and emits in the orange-range of the light spectrum. Changes in the ratio between aggregates and monomers gives an arbitrary measure of the changes in mitochondrial potential. Therefore, PBTs were resuspended in PBS with 2 µM dye and incubated for 30 min at 37 °C, 5 % CO_2_. Data are represented as the ratio of the MFI of the aggregates (orange fluorescence) to the one of the monomers (green fluorescence).

For the determination of intracellular ROS levels, 5 µM CellROX™ Green Reagent (ThermoFisher Scientific, Waltham, Massachusetts) was added to the cells and incubated at 37 °C, 5 % CO_2_ for 30 min.

Total neutral lipid content was analyzed by staining PBTs for 15 min with 2 µM BODIPY™493/503 (ThermoFisher Scientific, Waltham, Massachusetts) at 37 °C, 5 % CO_2_.

To monitor fatty acid uptake, cells were incubated with 1 µM BODIPY™ FL C_16_ (ThermoFisher Scientific, Waltham, Massachusetts) diluted in serum-free RPMI for 10 min at 4 °C.

### Flow cytometry

Monoclonal antibodies recognizing the following surface markers and molecules were used for flow cytometry: CCR7 (G043H7), CD3 (SK7), CD25 (M-A251), CD45RA (HI100), CD54 (LB-2), CD58 (TS2/9), CD69 (FN50), CD80 (L307.4), CD86 (2331), CD166 (3A6), CD318 (CUB1). 7-AAD was used to specifically stain dead cells. All antibodies were obtained from BD Bioscience (Franklin Lakes, New Jersey), eBioscience (Affymetrix, Santa Clara, California) or BioLegend (San Diego, California).

### Statistics

Statistical analyses were performed with Prism 9 (GraphPad, San Diego, California) software. Values are expressed as mean ± SEM. Analysis of variance (ANOVA) and unpaired two-tailed Student *t* test were used to test significant differences between groups. Differences of *p* ≤ 0.05 were statistically significant (* *p* ≤ 0.05; ** *p* ≤ 0.01; *** *p* ≤ 0.001; **** *p* < 0.0001).

## Data availability statement

The original contributions presented in the study are included in the article/**Supplementary Material**. Further inquiries can be directed to the corresponding author.

## Ethics statement

This study was reviewed and approved by Ethics Committee of Heidelberg University (S-135/2019). The patients/participants provided their written informed consent to participate in this study.

## Author contributions

Conceptualization: CO, YS. Methodology: CO, KB, EB, MS-C, JS-B. Investigation: CO, KB, BJ. Visualization: CO, KB. Funding acquisition: YS. Project administration: YS. Supervision: YS. Resources: YS, BN, MS-C, KS. Writing – original draft: CO, KB, YS. All authors contributed to the article and approved the submitted version.

## Funding

This work was supported by a grant of the German Research Foundation DFG TRR156-246807620 project B04. For the publication fee we acknowledge financial support by the Deutsche Forschungsgemeinschaft within the funding programme “Open Access publication cost” as well as by Heidelberg University.

## Acknowledgments

We thank Ralph Röth (nCounter Core Facility, Heidelberg) for his help during the planning of the customized *Elements codeset* and for performing the mRNA gene expression analysis. We thank Dr. Karel Klika (German Cancer Research Center, Heidelberg) for his assistance with NMR Spectroscopy.

## Conflict of interest

MMSC received scientific funding from Novartis and Pfizer.

The authors remaining declare that the research was conducted in the absence of any commercial or financial relationships that could be construed as a potential conflict of interest.

## Publisher’s note

All claims expressed in this article are solely those of the authors and do not necessarily represent those of their affiliated organizations, or those of the publisher, the editors and the reviewers. Any product that may be evaluated in this article, or claim that may be made by its manufacturer, is not guaranteed or endorsed by the publisher.
