# Supplementary material for: Keratinocyte-induced costimulation of human T cells through CD6 - but not CD2 - activates mTOR and prevents oxidative stress
Source: Front Immunol. 2022 Oct 24;13:1016112. doi: 10.3389/fimmu.2022.1016112 (PMC9639098; doi:10.3389/fimmu.2022.1016112)
Supplement: Supplementary file 1 [file DataSheet_1.pdf]

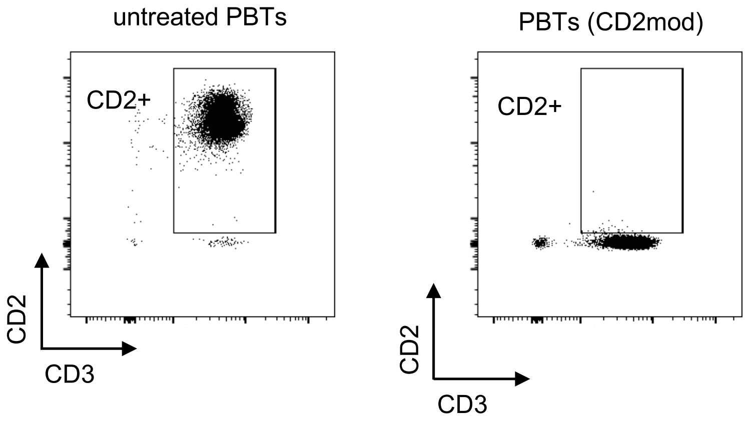

Figure S1. CD2 staining after treatment with CD2-modulating antibody. Representative dot plots of surface expression of CD3 and CD2 on untreated peripheral blood T cells (PBTs) and CD2 downmodulated (time point: 24 h) PBTs (CD2mod).

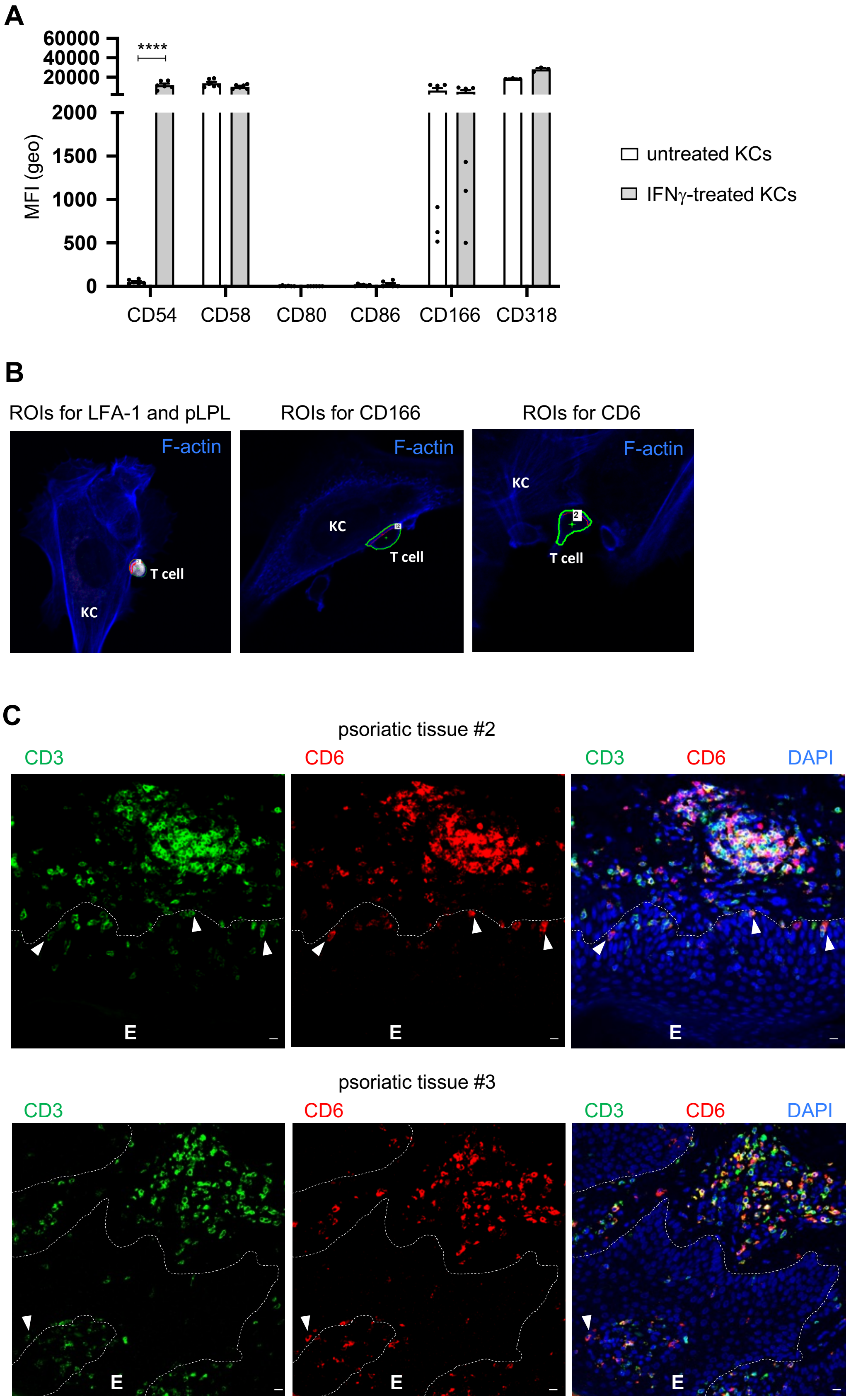

Figure S2. CD166 and CD6 accumulate at the contact zone between primary human keratinocytes and PBTs. A: Statistical evaluation of flow cytometric analysis of costimulatory receptors on untreated KCs (black line, white filling) and KCs pretreated with IFNγ for 24 h (black line, grey filling) (n ≥ 3). Data is represented as

mean ± SEM. ****=p<0.0001; ***=p<0.001; **=p<0.01. **B:** Representative picture of IFNγ-pretreated KCs and T cells with Region of interest (ROI) of contact zone (1, red) or whole cell (2, green) which was used to calculate the polarization index (PI) of the respective signals. **C:** Punch biopsies of psoriatic skin were stained for CD3 and CD6. Representative immunofluorescence staining of punch biopsies of skin lesions of psoriasis patient using Opal-4-color IHC kit (CD3 (green), CD6 (red), DAPI (blue)). Dashed white line represents the border of epidermis (E) to dermis or hair follicle, respectively. White triangles highlight epidermal T cells highly expressing CD6.

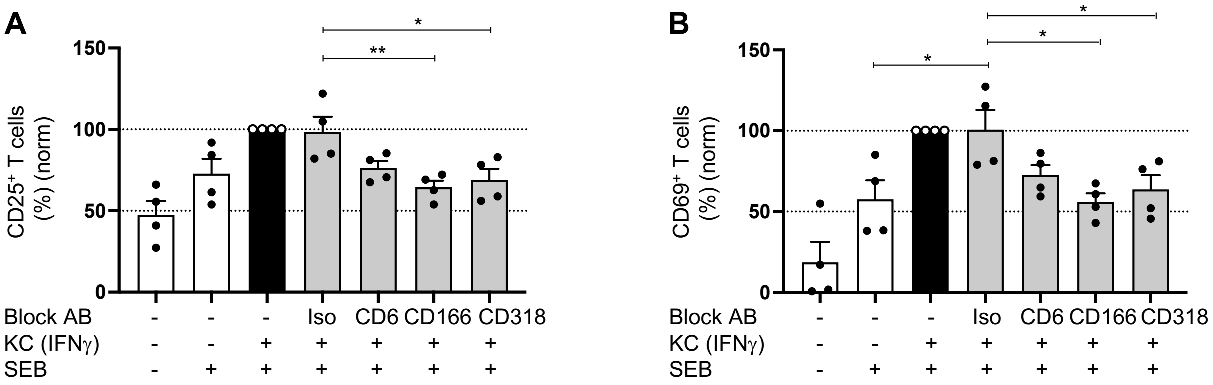

Figure S3. Effect of blocking antibodies against CD6, CD166 or CD318 on T cell activation marker expression. $CD3^{+}$ PBTs were cultured for 24 h with untreated keratinocytes (KCs) (white bars) or IFNγ-pretreated KCs (black and grey bars), loaded with (+) or without (-) SEB and then analyzed by flow cytometry. **A-B:** Statistical evaluation of the effect of blocking antibodies against costimulatory receptors (CD6, CD166, CD318) or isotype control antibodies (iso) on **(A)** CD25 and **(B)** CD69 expression (n = 4 individual T cell donors). Data was normalized to PBTs cultured with IFNγ-pretreated KCs loaded with SEB (black bar). Data is represented as mean ± SEM. **=p<0.01; *=p<0.05.

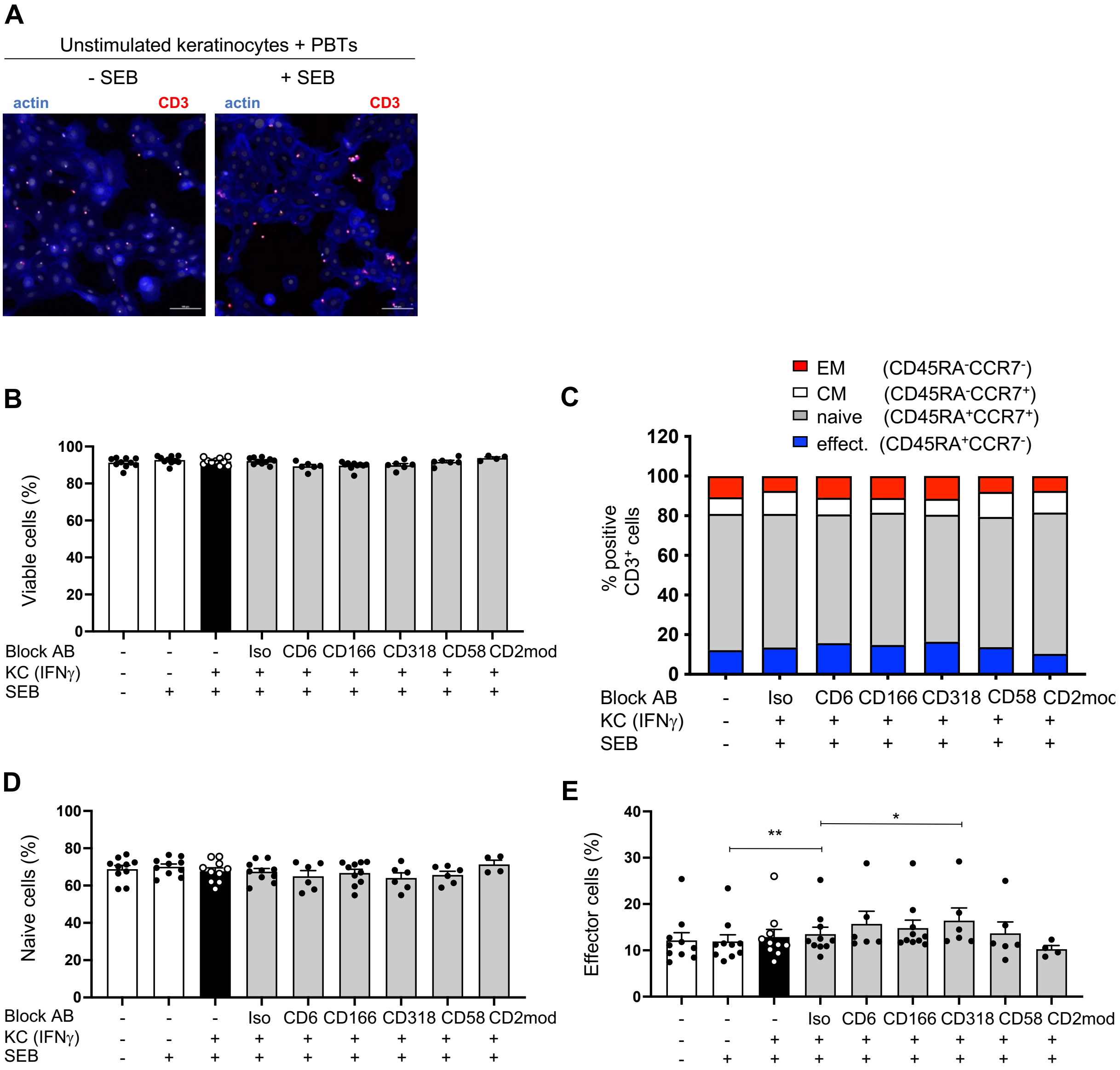

Figure S4. T cell adhesion to unstimulated KCs and T cell viability as well as subset distribution upon blocking the CD6/CD166/CD318 axis. A: Adhesion of $CD3^+$ PBTs to unstimulated KCs without or with SEB-loading. Cells were cocultured for 4 h and analyzed by confocal microscopy (20x objective; NA = 0.75). Representative immunofluorescence stainings for F-actin (blue) and CD3 (red) are shown.

B-D: $CD3^+$ T cells were cultured for 72 h with untreated KCs (white bars) or IFNγ-pretreated KCs (black and grey bars), loaded with (+) or without (-) SEB and then analyzed by flow cytometry. Statistical evaluation of the effect of blocking antibodies or isotype control antibodies (iso) on **(B)** T cell viability assessed by 7-AAD staining ($n \geq 4$ individual T cell donors); **(C-E)** percentages of T cell populations **(D)** naïve ($CD45RA^+CCR7^+$) and **(E)** effector (effect., $CD45RA^+CCR7^-$) T cells ($n \geq 4$ individual T cell donors). Data is represented as mean ± SEM. **=p<0.01; *=p<0.05.

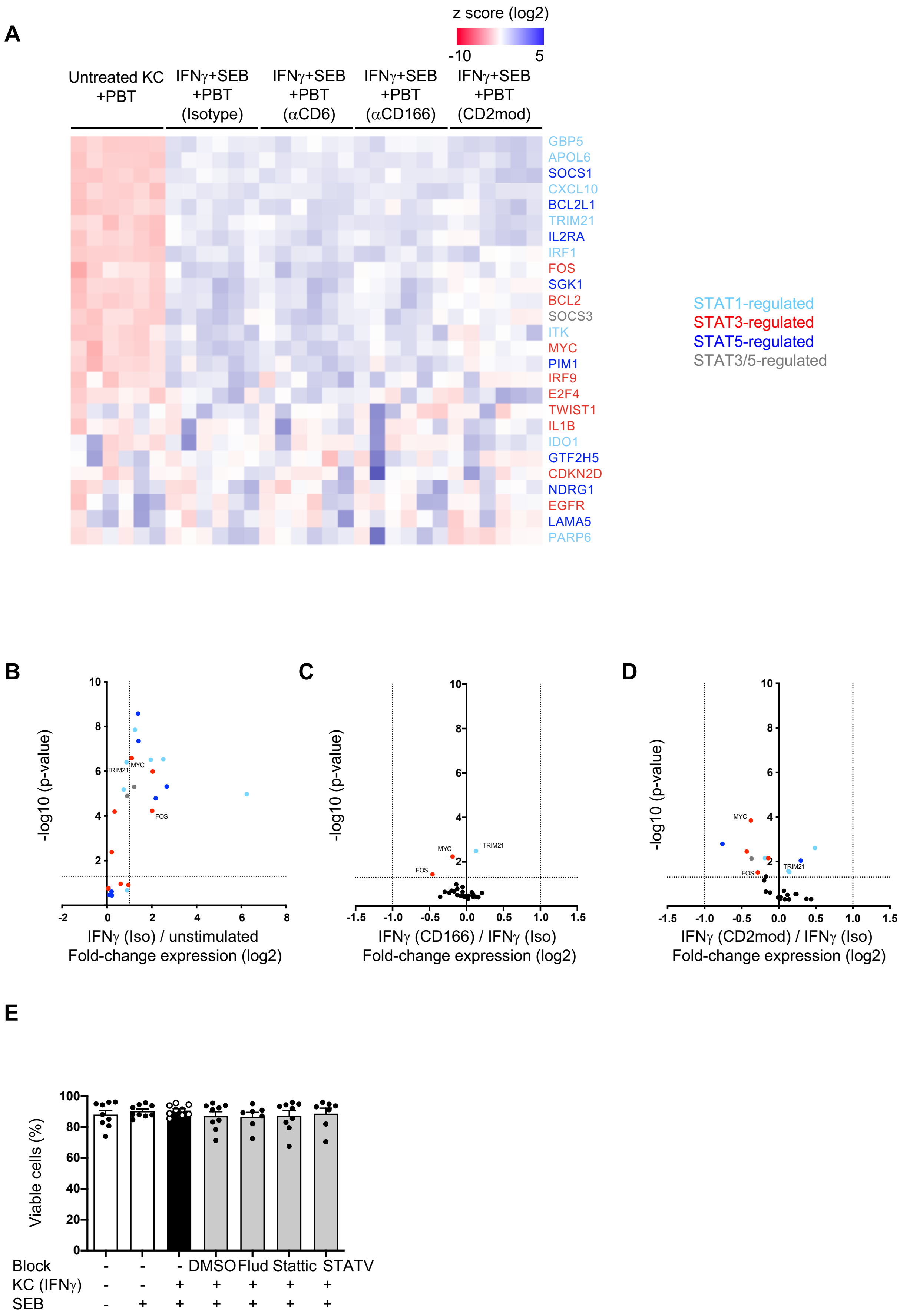

Figure S5. STAT-signaling during KC-dependent T cell activation. CD3⁺ PBTs were cultured with untreated KCs or IFNγ-pretreated KCs, loaded with SEB and then

analyzed by Nanostring nCounter GEx (mRNA expression analyzed by a Nanostring customized *Elements* Panel) after 4 h or flow cytometry (cell viability) after 72 h. **A**: Heat map of regulated genes in $CD3^+$ PBTs in a 4 h coculture (each column of either 'untreated KCs + PBTs' or 'IFNγ-pretreated + PBTs' with blocking antibodies or isotype control antibodies represents data points derived from T cells from one individual donor, 6 donors in total). STAT1- (light blue), STAT3- (red), STAT-5 (dark blue) or STAT3/5-regulated genes (grey) are labeled with distinct colors.

B-D: Volcano plots of the fold-change in mRNA code counts (log-2 transformed) plotted against significance of the change (-$\log_{10}$ transformed p value). Fold-changes were calculated from the mRNA code counts isolated from PBTs of 6 individual donors after a 4 h coculture. **(B)** Fold-changes of PBTs cultured with IFNγ-pretreated and SEB loaded KCs in the presence of isotype control antibodies compared to the culture with untreated KCs. **(C)** Fold-changes of PBTs cultured with IFNγ-pretreated and SEB-loaded KCs in the presence of blocking antibody against CD166 compared to the culture with IFNγ-pretreated KCs in the presence of isotype control antibodies. **(D)** Fold-changes of PBTs cultured with IFNγ-pretreated and SEB-loaded KCs in the presence of CD2-modulating antibody (CD2mod) compared to the culture with IFNγ-pretreated KCs in the presence of isotype control antibodies. **E**: Percentage of viable PBTs (7-AAD negative) after coculture for 72 h with untreated KCs or IFNγ-pretreated KCs loaded with SEB either in the presence or absence of DMSO, Fludarabine (Flud), Stattic or STATV ($n \geq 7$ individual T cell donors). Data is represented as mean ± SEM.

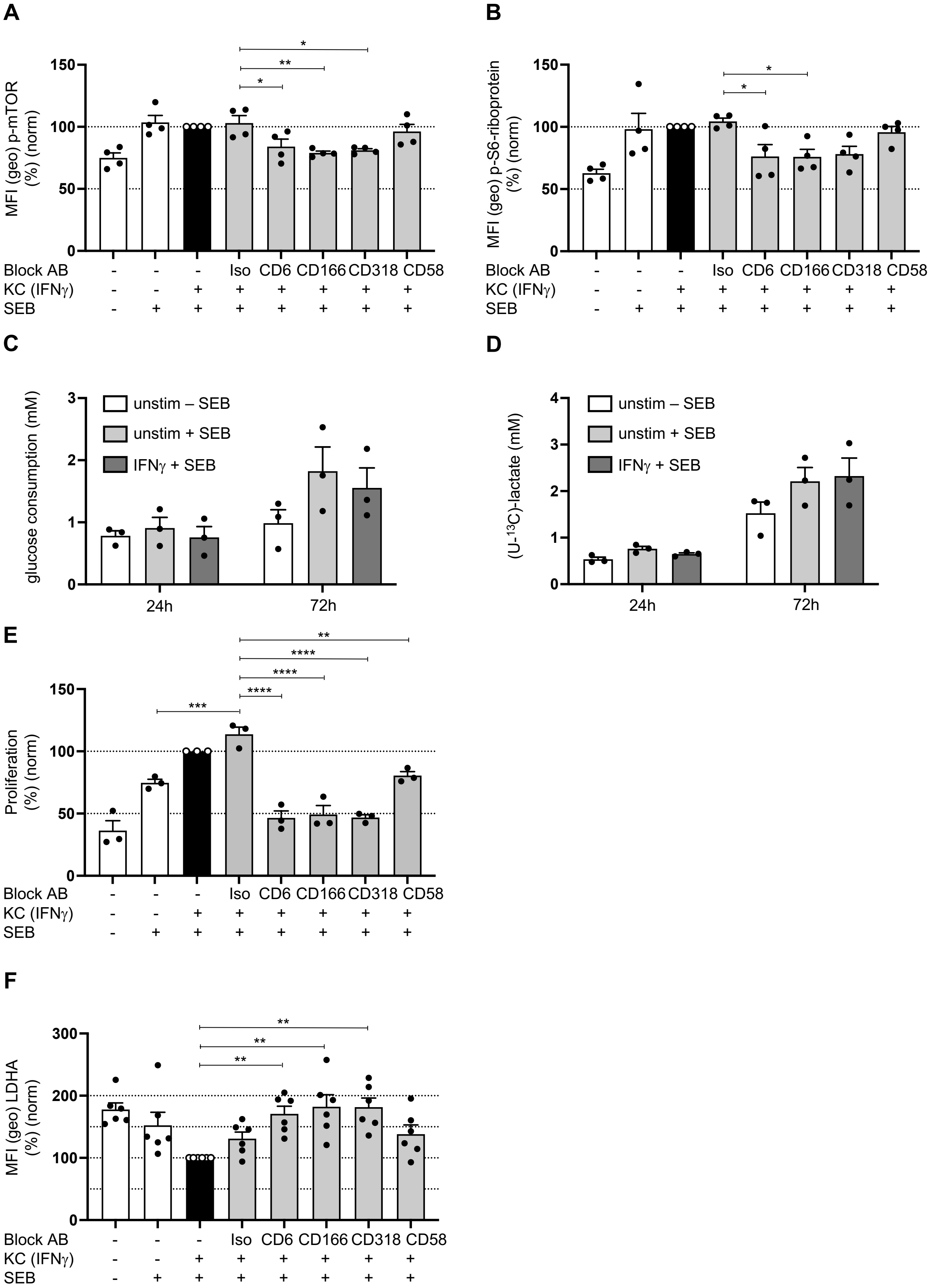

Figure S6. Metabolic changes induced by KC-dependent T cell activation and the influence of CD6/CD166/CD318 blockade. CD3^{+} PBTs were cultured with untreated KCs (white bars) or IFNγ-pretreated KCs (black and grey bars), loaded with SEB and the effects of blocking antibodies against CD6, CD166, CD318 or CD58 or isotype

control antibodies (iso) were analyzed by flow cytometry**. A-B**: mTOR activation after 72 h coculture. **(A)** MFI of phosphorylated mTOR and **(B)** MFI of phosphorylated S6-riboprotein (n = 4 individual T cell donors). **C-D:** Metabolism of isotopically labeled [U-^{13}C]-glucose was analyzed in the supernatant of PBTs cultured for 24 h or 72 h with fixed untreated KCs (white bars) or IFNγ-pretreated and SEB-loaded KCs (black and grey bars) by ^{1}H-NMR. **(C)** Statistical evaluation of glucose consumption and **(D)** concentration of [U-^{13}C]-lactate (n = 3 individual T cell donors)**. E**: T cell proliferation after 72 h coculture assessed by CFDA dilution assay. Statistical evaluation (n = 3 individual T cell donors). **F**: Intracellular LDHA expression (MFI) in PBTs cocultured for 72 h. Statistical evaluation (n = 6 individual T cell donors). Data was normalized to PBTs cultured with IFNγ-pretreated KCs loaded with SEB (black bar). Data is represented as mean ± SEM. ****=p<0.0001; ***=p<0.001; **=p<0.01; *=p<0.05.

Table S1. ***Elements codeset*** **for nCounter analysis.**

Gene	Accession	Targeted Region	Target Sequence
GAPDH	NM_001256799.1	387-486	GAACGGGAAGCTTGTCATCAATGGAAATCCCATCAC CATCTTCCAGGAGCGAGATCCCTCCAAAATCAAGTG GGGCGATGCTGGCGCTGAGTACGTCGTG
TBP	NM_001172085.1	588-687	ACAGTGAATCTTGGTTGTAAACTTGACCTAAAGACC ATTGCACTTCGTGCCCGAAACGCCGAATATAATCCC AAGCGGTTTGCTGCGGTAATCATGAGGA
TUBB	NM_178014.2	1956-2055	TTCTAAGTATGTCCATTTCCCATCTCAGCTTCAAGG GAGGTGTCAGCAGTATTATCTCCACTTTCAATCTCC CTCCAAGCTCTACTCTGGAGGAGTCTGT
RPL19	NM_000981.3	316-415	CCAATGCCCGAATGCCAGAGAAGGTCACATGGATG AGGAGAATGAGGATTTTGCGCCGGCTGCTCAGAAG ATACCGTGAATCTAAGAAGATCGATCGCCA
POLRA	NM_000937.2	3776-3875	TTCCAAGAAGCCAAAGACTCCTTCGCTTACTGTCTT CCTGTTGGGCCAGTCCGCTCGAGATGCTGAGAGAG CCAAGGATATTCTGTGCCGTCTGGAGCAT
EEF1G	NM_001404.4	1151-1250	CTTCATGAGCTGCAATCTCATCACTGGAATGTTCCA GCGACTGGACAAGCTGAGGAAGAATGCCTTCGCCA GTGTCATCCTTTTTGGAACCAACAATAGC
OAZ1	NM_004152.2	314-413	GGTGGGCGAGGGAATAGTCAGAGGGATCACAATCT TTCAGCTAACTTATTCTACTCCGATGATCGGCTGAA TGTAACAGAGGAACTAACGTCCAACGACA
IL2RA	NM_000417.1	1001-1100	CTTGGTAAGAAGCCGGGAACAGACAACAGAAGTCA TGAAGCCCAAGTGAAATCAAAGGTGCTAAATGGTCG CCCAGGAGACATCCGTTGTGCTTGCCTGC
IL1B	NM_000576.2	841-940	GGGACCAAAGGCGGCCAGGATATAACTGACTTCAC CATGCAATTTGTGTCTTCCTAAAGAGAGCTGTACCC AGAGAGTCCTGTGCTGAATGTGGACTCAA
CXCL10	NM_001565.2	462-561	GCCATAATTGTTCTTAGTTTGCAGTTACACTAAAAGG TGACCAATGATGGTCACCAAATCAGCTGCTACTACT CCTGTAGGAAGGTTAATGTTCATCATC
BCL2	NM_000657.2	6-105	GTGAAGCAGAAGTCTGGGAATCGATCTGGAAATCC TCCTAATTTTTACTCCCTCTCCCCGCGACTCCTGATT CATTGGGAAGTTTCAAATCAGCTATAAC
GBP5	NM_052942.3	1956-2055	ATTACAGACTGACCAGGCTCTCACAGAGACGGAAA AAAAGAAGAAAGAGGCACAAGTGAAAGCAGAAGCT GAAAAGGCTGAAGCGCAAAGGTTGGCGGCG
IDO1	NM_002164.5	370-469	ATCACCATGGCATATGTGTGGGGCAAAGGTCATGG AGATGTCCGTAAGGTCTTGCCAAGAAATATTGCTGT TCCTTACTGCCAACTCTCCAAGAAACTGG
IRF1	NM_002198.2	16-115	TTAGTCGAGGCAAGACGTGCGCCCGAGCCCCGCC GAACCGAGGCCACCCGGAGCCGTGCCCAGTCCAC GCCGGCCGTGCCCGGCGGCCTTAAGAACCCGG
SOCS1	NM_003745.1	1026-1125	TTAACTGTATCTGGAGCCAGGACCTGAACTCGCACC TCCTACCTCTTCATGTTTACATATACCCAGTATCTTT GCACAAACCAGGGGTTGGGGGAGGGTC
SOCS3	NM_003955.3	1871-1970	GGAGGATGGAGGAGACGGGACATCTTTCACCTCAG GCTCCTGGTAGAGAAGACAGGGGATTCTACTCTGT GCCTCCTGACTATGTCTGGCTAAGAGATTC
APOL6	NM_030641.3	9056-9155	GGAACAAAGTTAATTGGTTCCAGAGATTCAAAGCCA GAGTTGCTGTCAGTTCATTGGTAGAGATGCCATCAC TGGGCAAGTGTTCTGAAAACATCTTATC
BCL2L1	NM_138578.1	1561-1660	CTAAGAGCCATTTAGGGGCCACTTTTGACTAGGGAT TCAGGCTGCTTGGGATAAAGATGCAAGGACCAGGA CTCCCTCCTCACCTCTGGACTGGCTAGAG
CDKN2D	NM_001800.3	871-970	CTTTCTCTTCTTGTTTCTCCTGCCCACTGCTGCAGTA GGGGAGGAGCACAGTTTGTGGCTTATAGGTGTTGG TTTTGGGGGTGTGAGTGTTTGGGGGACG
E2F4	NM_001950.3	221-320	GCCAGAAGCGGCGGATTTACGACATTACCAATGTTT TGGAAGGTATCGGGCTAATCGAGAAAAAGTCCAAG AACAGCATCCAGTGGAAGGGTGTGGGGCC

EGFR	NM_201282.1	1355-1454	ACATCCTGCCGGTGGCATTTAGGGGTGACTCCTTC<br>ACACATACTCCTCCTCTGGATCCACAGGAACTGGAT<br>ATTCTGAAAACCGTAAAGGAAATCACAGG
FOS	NM_005252.2	1476-1575	ACTCAAGTCCTTACCTCTTCCGGAGATGTAGCAAAA<br>CGCATGGAGTGTGTATTGTTCCCAGTGACACTTCAG<br>AGAGCTGGTAGTTAGTAGCATGTTGAGC
GTF2H5	NM_207118.2	661-760	TCAGTTAGAAACGTCATAGATTTGCTGTTTGAATATG<br>CCAAGGTGGGGACTTAGACATTATGTACGTCTCACA<br>AATCCTACCTGCATACCAGTCAGCTCT
ID1	NM_002165.2	346-445	CTGCCCCAGAACCGCAAGGTGAGCAAGGTGGAGAT<br>TCTCCAGCACGTCATCGACTACATCAGGGACCTTCA<br>GTTGGAGCTGAACTCGGAATCCGAAGTTG
IRF9	NM_006084.4	386-485	GCACTCAACAAGAGTTCTGAATTTAAGGAGGTTCCT<br>GAGAGGGGCCGCATGGATGTTGCTGAGCCCTACAA<br>GGTGTATCAGTTGCTGCCACCAGGAATCG
ITK	NM_005546.3	3431-3530	GCCAGTAAAGAAGTCAGTATAGAACCACTAGCGAAT<br>AGTGTTGCTCTGGCACAGACCACTGTGGTTGATGG<br>CATGGCCCTCCAACTTGGAATAGGATTTT
LAMA5	NM_005560.3	788-887	GCCATGAATTTCTCCTACTCGCCGCTGCTACGTGAG<br>TTCACCAAGGCCACCAACGTCCGCCTGCGCTTCCT<br>GCGTACCAACACGCTGCTGGGCCATCTCA
MYC	NM_002467.3	1611-1710	TCGGACACCGAGGAGAATGTCAAGAGGCGAACACA<br>CAACGTCTTGGAGCGCCAGAGGAGGAACGAGCTAA<br>AACGGAGCTTTTTTGCCCTGCGTGACCAGA
NDRG1	NM_001135242.1	2721-2820	TGGGGGTGGAATTGGGGTTACTCGATGTAAGGGAT<br>TCCTTGTTGTTGTGTTGAGATCCAGTGCAGTTGTGA<br>TTTCTGTGGATCCCAGCTTGGTTCCAGGA
PARP6	NM_020214.2	1319-1418	TGGTGTGTGATGAGCAGCATGTCTTCCAAAATGGAT<br>CTATGCTGAAGCCAGCTGTCTGTACTCGTGAACTAT<br>GCGTTTTCTCCTTCTACACACTGGGCGT
PIM1	NM_002648.2	1631-1730	CTTCATCATGAGTTCTGCTGAATGCCGCGATGGGTC<br>AGGTAGGGGGGAAACAGGTTGGGATGGGATAGGA<br>CTAGCACCATTTTAAGTCCCTGTCACCTCT
SGK1	NM_005627.2	1791-1890	GTGTGAACCGTCGTGTGAGTGTGGTATGCCTGATC<br>ACAGATGGATTTTGTTATAAGCATCAATGTGACACTT<br>GCAGGACACTACAACGTGGGACATTGTT
TRIM21	NM_003141.3	1191-1290	AGAGACTCTGTGCGCAGGAAGGGGCACTTTTTGCT<br>TAGTTCCAAGAGTGGCTTCTGGACAATTTGGTTGTG<br>GAACAAACAAAAATATGAGGCTGGCACCT
TWIST1	NM_000474.3	36-135	CAACTCCCAGACACCTCGCGGGCTCTGCAGCACCG<br>GCACCGTTTCCAGGAGGCCTGGCGGGGTGTGCGT<br>CCAGCCGTTGGGCGCTTTCTTTTTGGACCTC
